# Supplementary material for: A linear nonribosomal octapeptide from Fusarium graminearum facilitates cell-to-cell invasion of wheat
Source: Nat Commun. 2019 Feb 25;10:922. doi: 10.1038/s41467-019-08726-9 (PMC6389888; doi:10.1038/s41467-019-08726-9)
Supplement: Supplementary file 1 — Supplementary Information [file 41467_2019_8726_MOESM1_ESM.pdf]

Supplementary Information

A Linear Nonribosomal Octapeptide from *Fusarium graminearum* Facilitates Cell-to-Cell Invasion of Wheat

Jia *et al*.

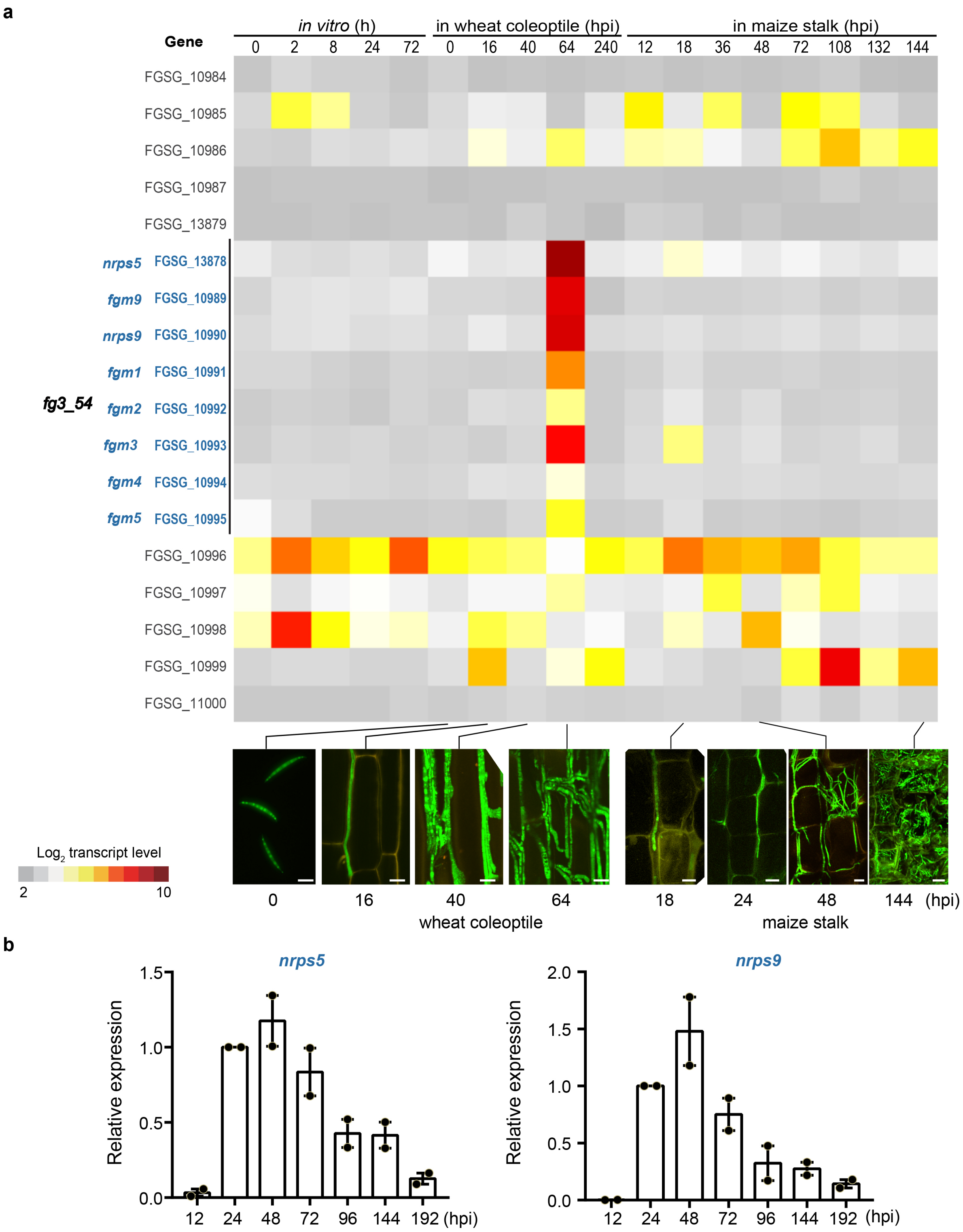

Supplementary Figure 1：**Expression analysis of *F. graminearum fg3_54* cluster and flanking genes. a,** Heat map presentation of expression data from previous laser microdissection-facilitated microarray analysis[1–3]. Plexdb: FG7, FG19. GEO: GSE53854. hpi: hours post inoculation. Representative images of *F. graminearum WT-AmCyan* at indicated time (below) during wheat coleoptile infection and maize stalk infection are from previous work[2,3]. **b,** Quantitative RT–PCR analysis of *nrps5* and *nrps9* during infection. Samples at 24 hpi were used as control. Data are means ± s.e.m. (n=2).

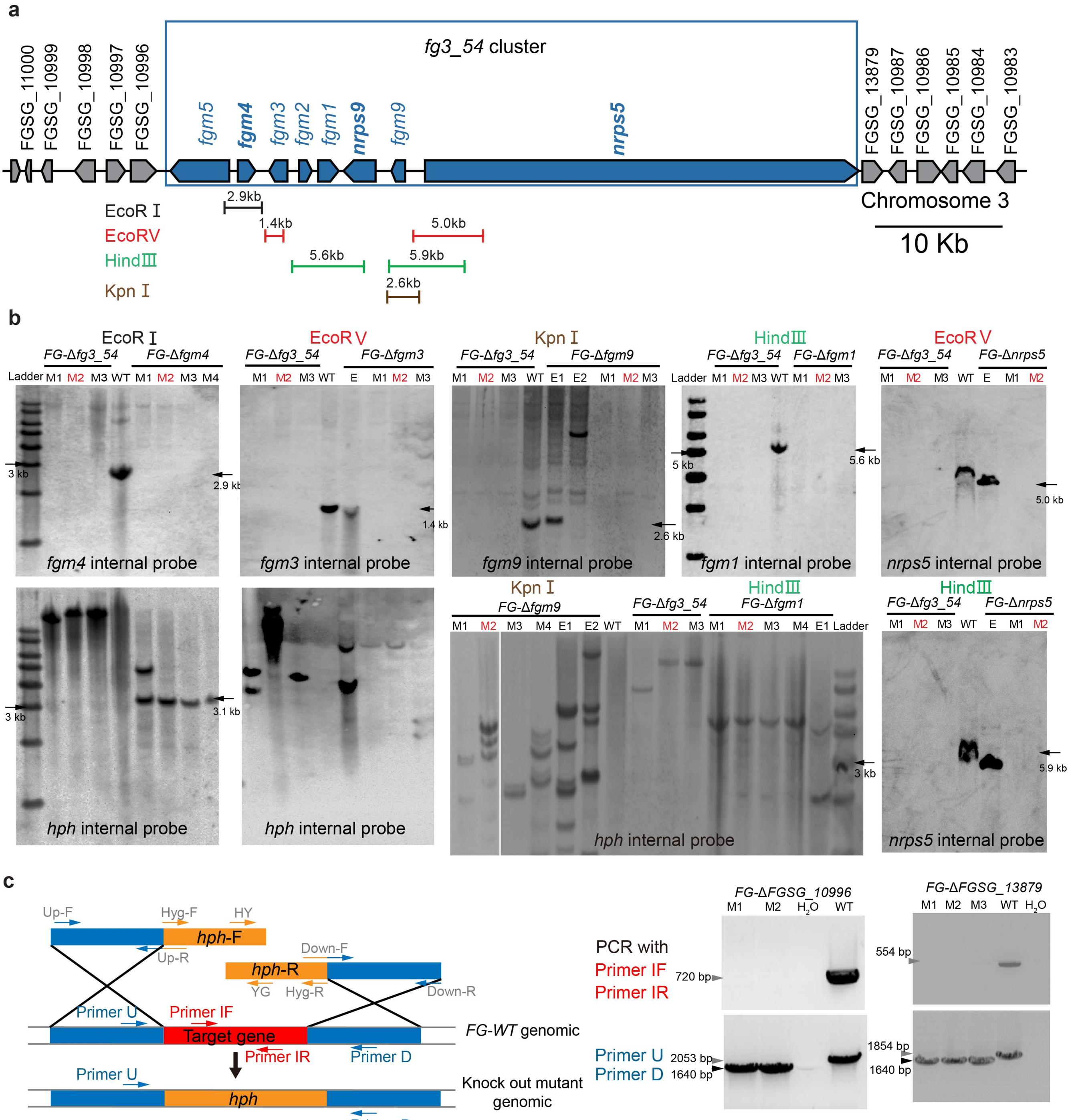

Supplementary Figure 2: Verification of *F. graminearum* gene deletion mutants. a, Restriction map of *fg3_54* gene cluster. **b,** Southern blot identification of gene deletion mutants with individual target gene probes and the replaced hygromycin B phosphotransferase gene (*hph*) probe using indicated restriction enzymes. **c,** Genomic DNA PCR verification of gene deletion mutants. Corresponding positions of the primers used for PCR are illustrated in the homologous recombination diagram. M1, M2, M3 and M4 represent independent transgenic lines of homologous recombination mutants. E, ectopic transformants. WT, wild-type strain PH-1. The line used for *fgm4* overexpression strain construction are in red.

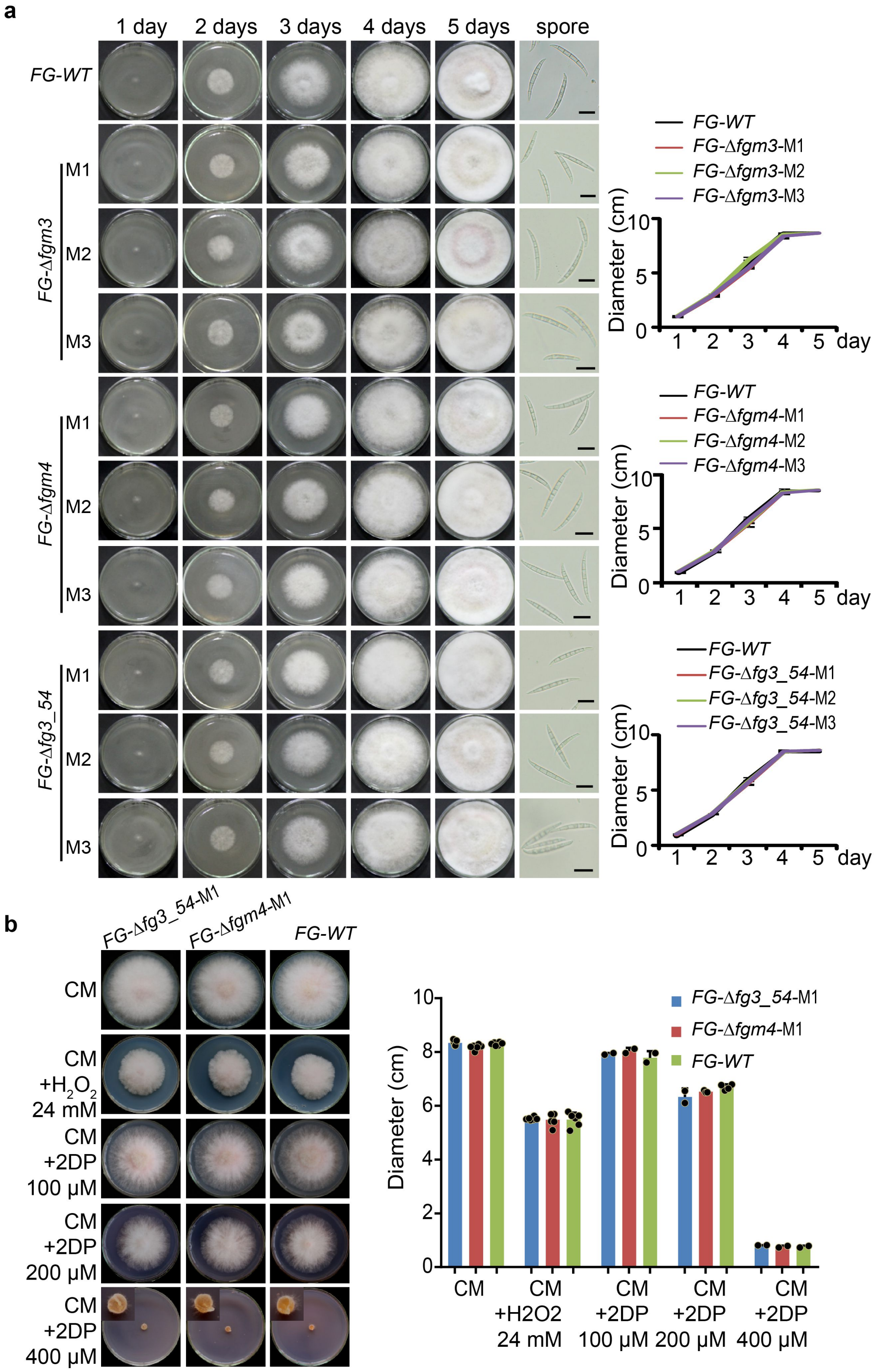

Supplementary Figure 3: *fg3_54*-related mutants showed no defect during in vitro culture. a, Representative pictures of *F. graminearum* strains growing on potato dextrose agar medium and spores from mung bean liquid medium. Scale bars represent 20 μm. **b,** Representative images of *F. graminearum* strains growing on complete medium (CM) supplemented with H_2O_2 or the iron chelator 2,2'-dipyridyl (2DP). Four-day-old cultures were photographed.

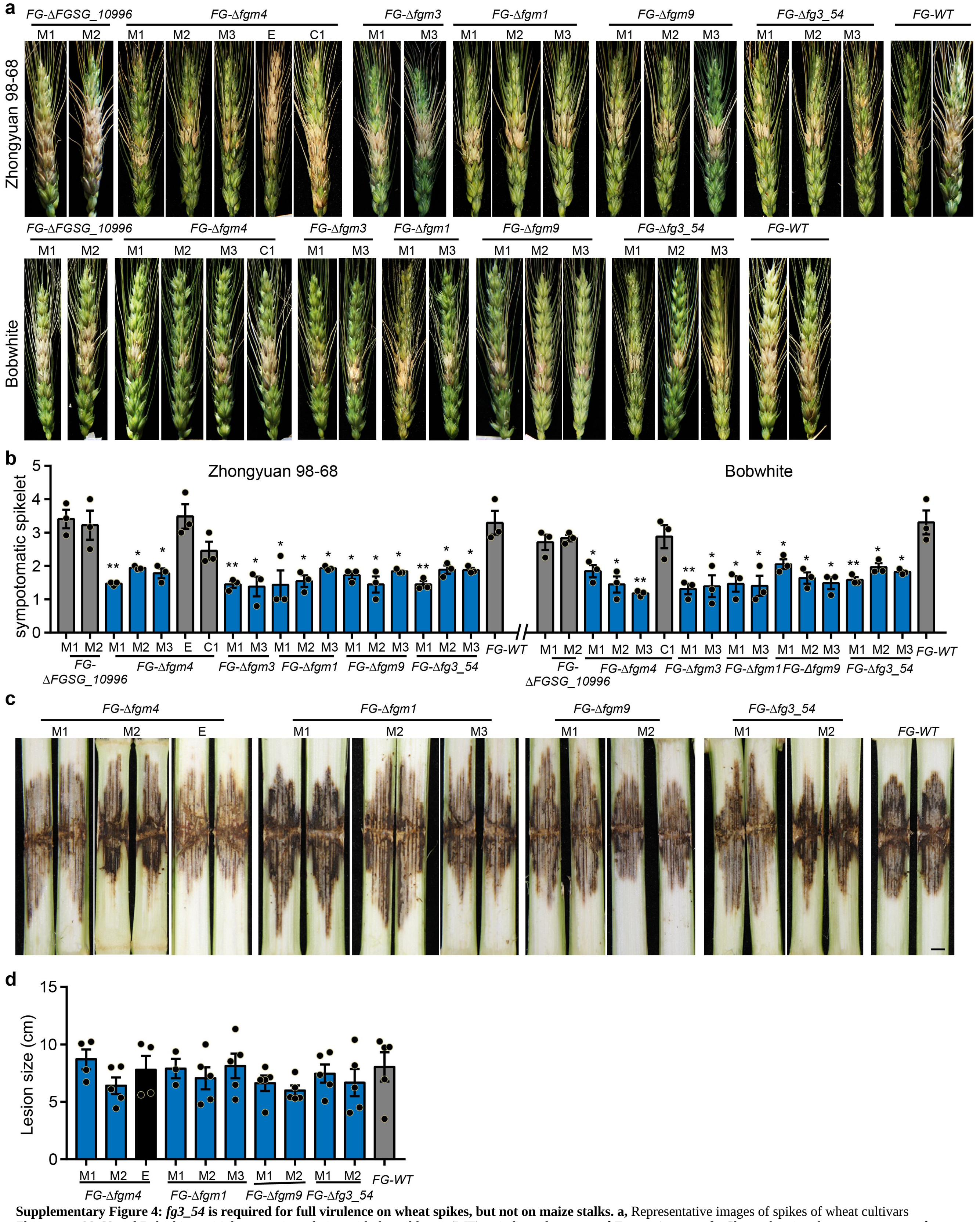

Supplementary Figure 4: *fg3_54* is required for full virulence on wheat spikes, but not on maize stalks. a, Representative images of spikes of wheat cultivars Zhongyuan 98-68 and Bobwhite at 14 days post inoculation with the wild type (WT) or indicated mutants of *F. graminearum*. **b,** Charts showing the measurements of symptomatic spikelet numbers at 14 dpi after inoculation with the indicated strains. Data are means±s.e.m. (n=3). Asterisks indicate statistically significant differences compared with the WT. **P< 0.01, *P<0.05 (Student's *t*-test). **c,** Representative images of maize (B73) stalks inoculated with indicated strains at 7 dpi. Scale bar represents 1 cm. **d,** Charts of lesion sizes in split stalks at 7 days after inoculated by indicated strains. Data are means±s.e.m. (n=3).

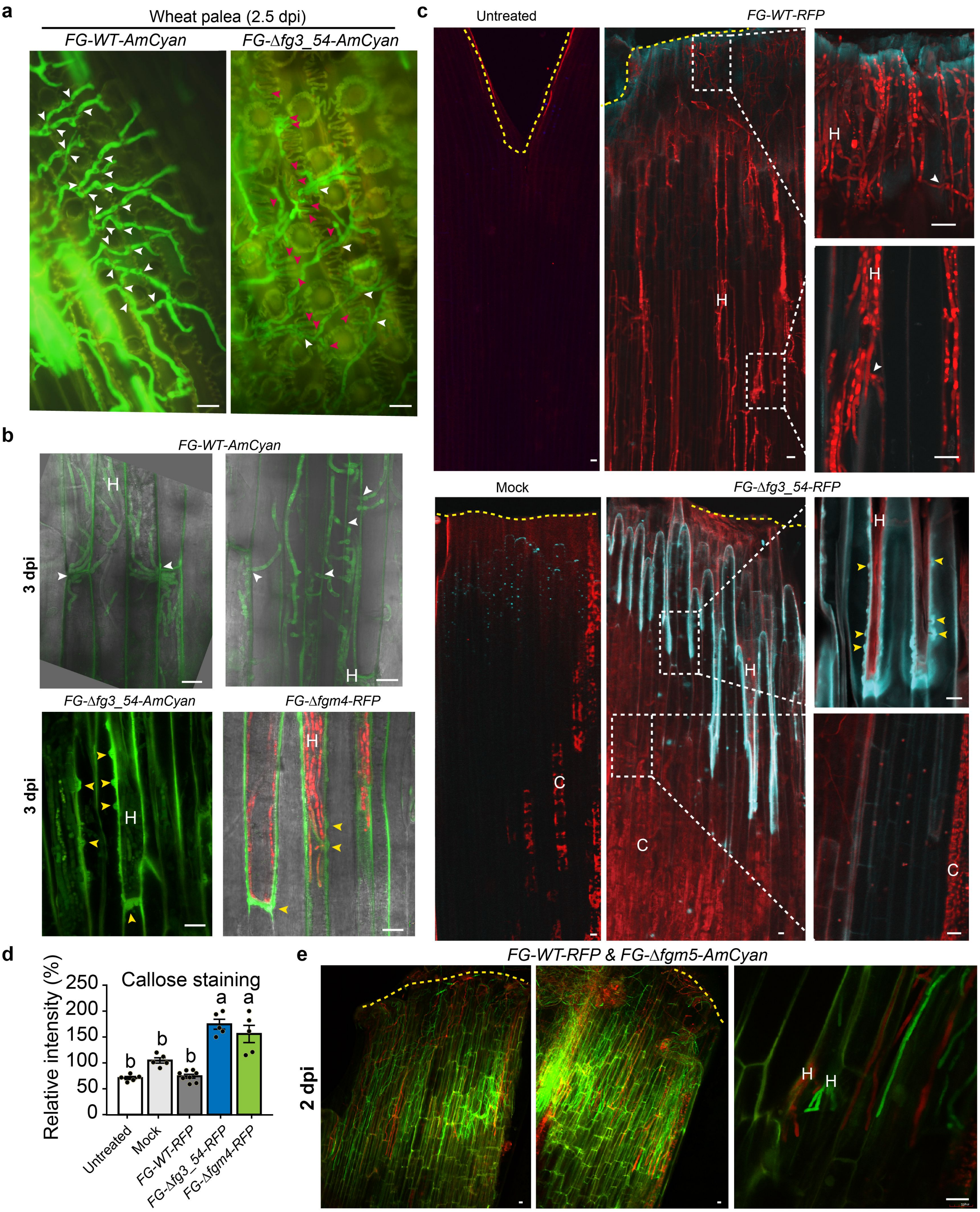

Supplementary Figure 5: Micrographs of *F. graminearum* growing inside wheat tissues. a, Growth of fluorescence-tagged fungal strains inside epidermis of palea at 2.5 dpi. White arrowheads indicate where hyphae penetrated host cell, and red arrowheads indicate where hyphae made unsuccessful attempts to penetrate host cell wall. **b,** Growth of fluorescence-tagged fungal strains inside coleoptiles at 3 dpi. Confocal fluorescent images were overlayed with DIC images. White arrowheads indicate where hyphae penetrated host cell wall, and yellow arrowheads indicate where plant cell wall thickened to form papillae-like structure. **c-d,** Coleoptiles were stained with aniline blue and the callose signal intensity was pictured (**c**) and quantified at 3 dpi (**d**). Yellow broken lines mark the wounded edge of coleoptiles where fungal spores were inoculated. Data are means±s.e.m. (n=3). Levels not connected by the same letters are significantly different ($P < 0.05$) in One-way ANOVA following Tukey's multiple comparison test ($F=31.84$, $df=4$, $P<0.0001$). **e,** The RFP-tagged WT and AmCyan-tagged mutant strains were co-inoculated on wounded coleoptiles and examined under a microscope at 2 dpi. Scale bars represent 20 µm.

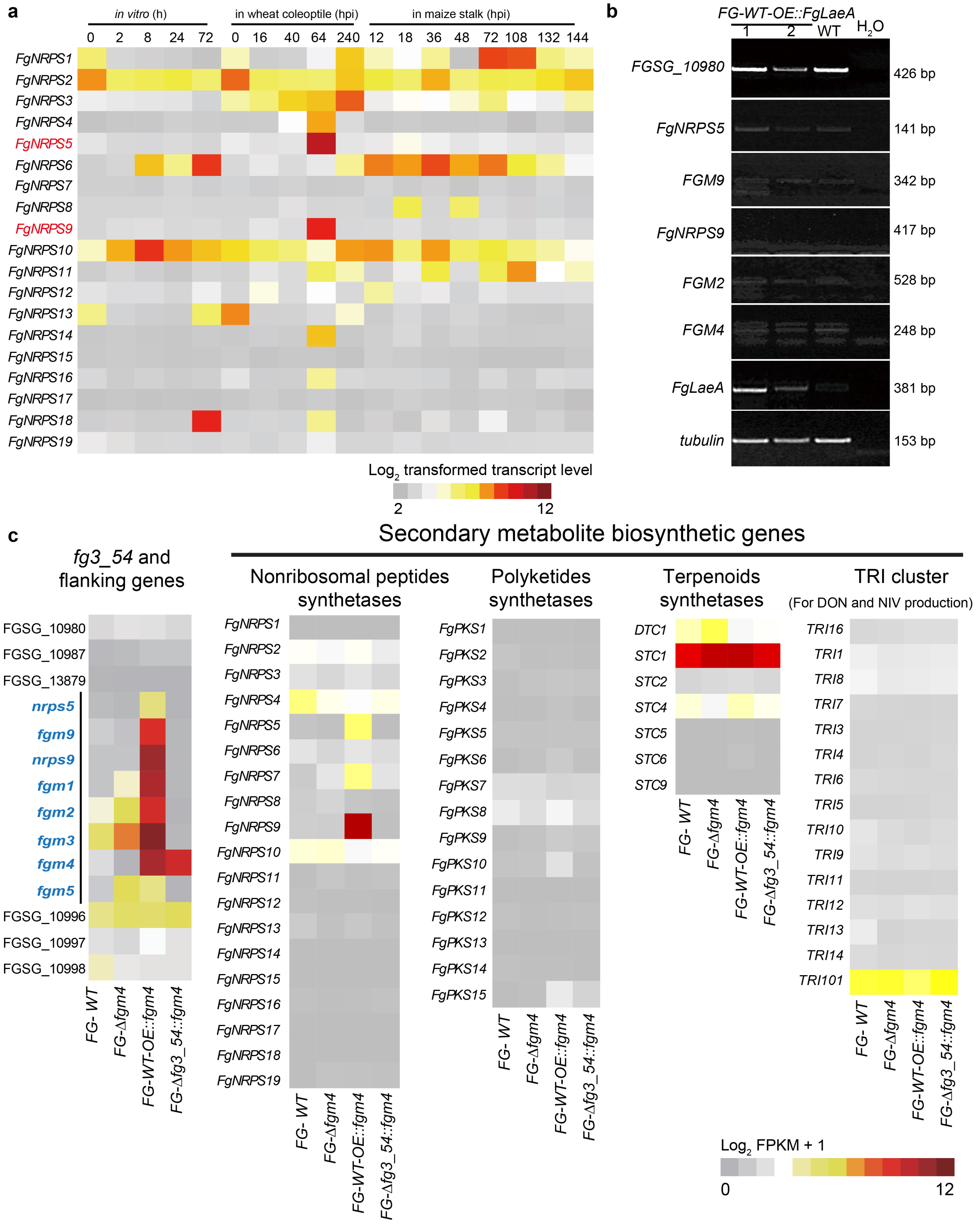

Supplementary Figure 6: Expression analysis of *fg3_54* and other secondary metabolic genes of *F. graminearum*. a, Heat map showing the expression of F. graminearum non-ribosomal peptide synthetase (NRPS) genes based on the microarray data[1–3]. **b,** RT-PCR analysis of *fg3_54* expression in the *FgLaeA* over-expression strains. 1 and 2 represent independent transgenic lines. Aerial mycelia of 3-day-old fungal colonies on V8 agar were used for RNA extraction. **c,** Heat map showing the expression of *F. graminearum fg3_54*-related, secondary metabolism structural genes and trichothecene biosynthetic pathway genes based on RNA-seq analysis of indicated fungal strains. PKS, polyketide synthase; TC, terpene cyclase; DTC, diterpene cyclase; STC, sesquiterpene cyclase.

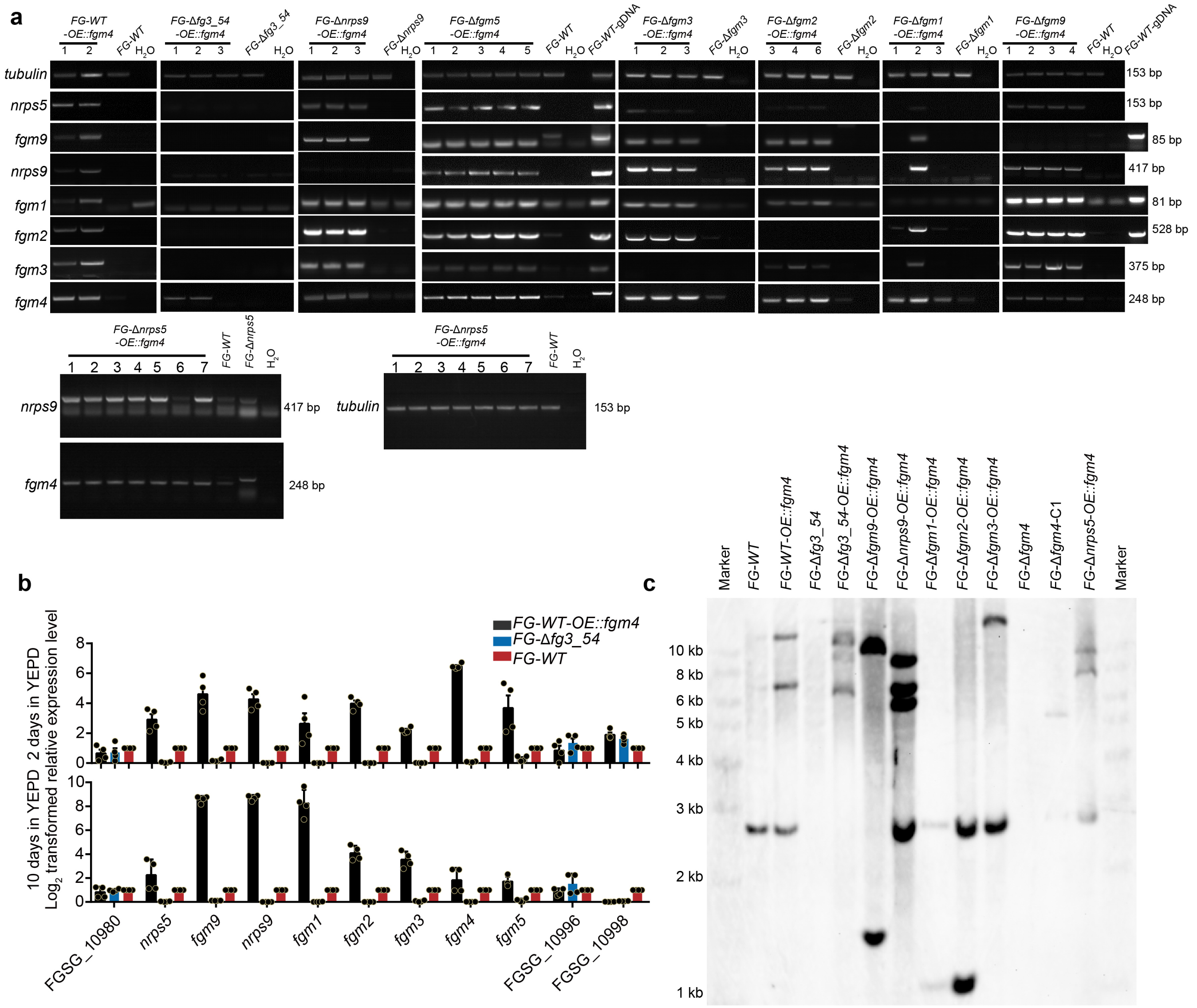

Supplementary Figure 7: RT-PCR (a), quantitative RT-PCR (b) and Southern blot (c) analysis of *fgm4* constitutive-expressing strains. a, RT-PCR analysis of *fg3_54* cluster gene expression. Numbers represent independent transgenic lines. Aerial mycelia of 3-day-old fungal colonies on V8 agar were used for RNA extraction. **b,** Quantitative RT–PCR analysis of *fg3_54* cluster gene expression. Mycelia growing in the YEPD media were used for RNA extraction. Data are means±s.e.m. (n=3). **c,** Southern blot analysis of representative *fgm4* constitutive-expressing strains. An intronless *fgm4* genomic DNA fragment is used as probe template.

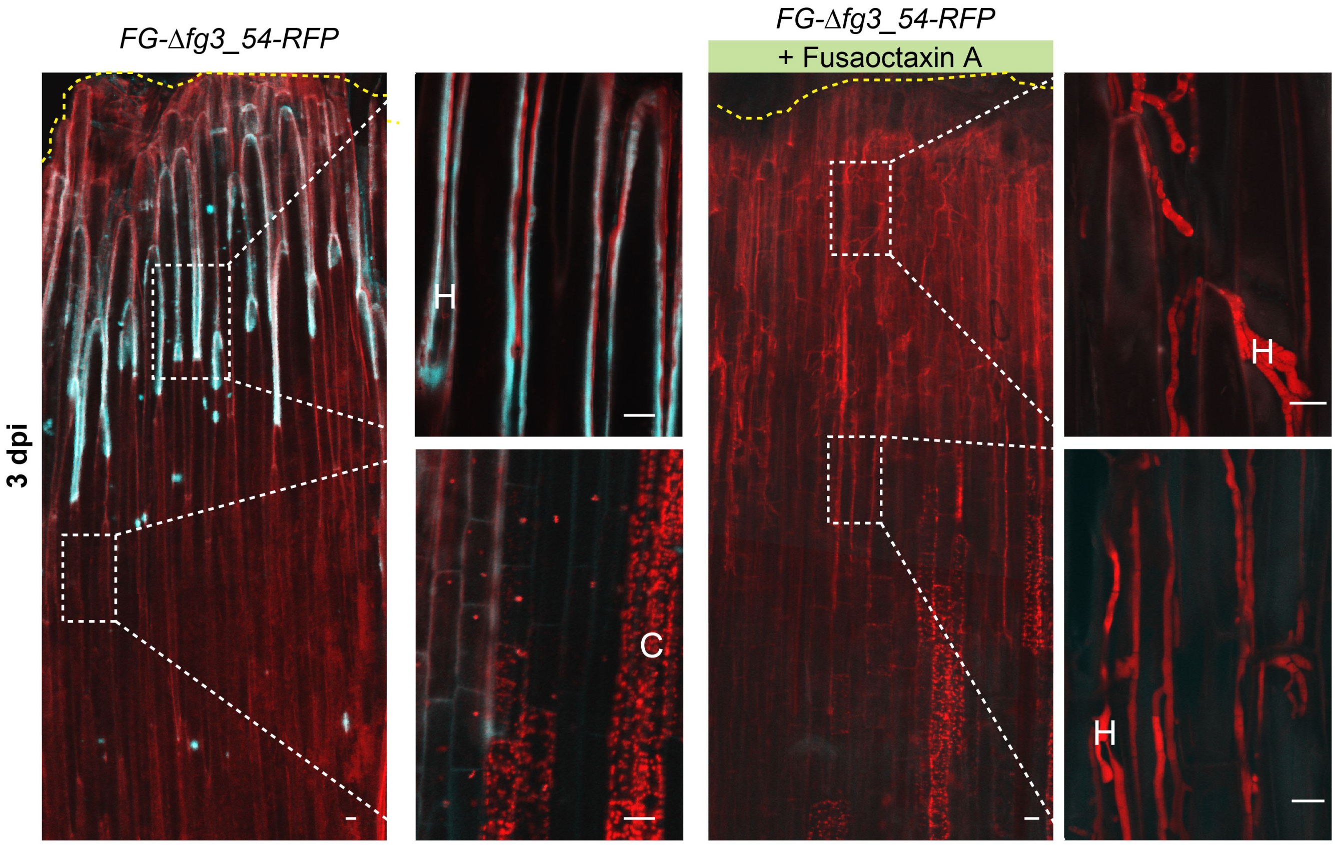

Supplementary Figure 8: Fluorescence microscopic analysis of wheat coleoptiles inoculated with *FG-Δfg3_54-RFP* strain and stained with aniline blue for callose at 3 dpi. White scale bars represent 20 µm. Images are overlays of CFP (callose staining) and RFP (fungal hyphae) channels.

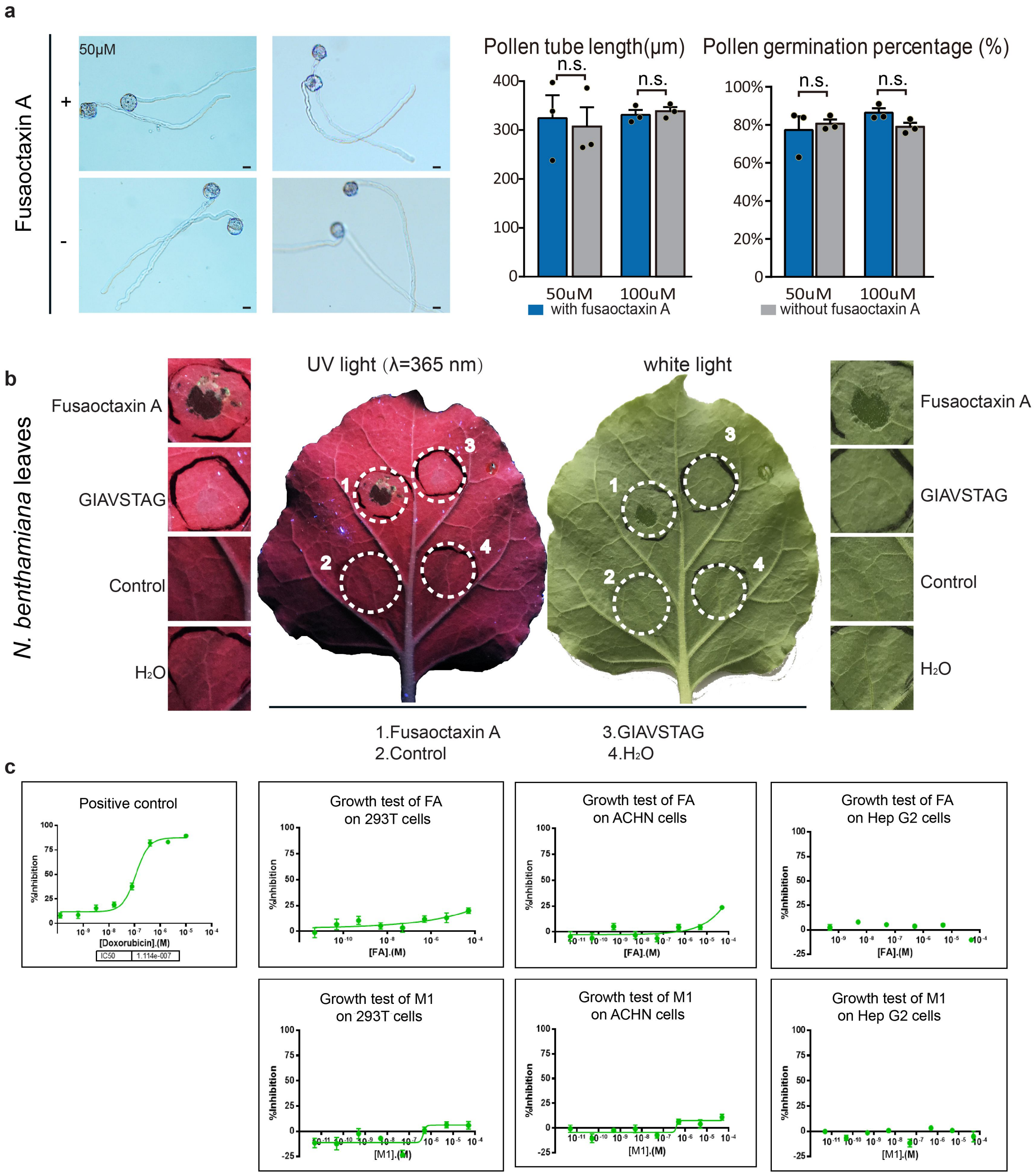

Supplementary Figure 9: Cytotoxicity assays of fusaoctaxin A. a, Representative pictures of tabacco pollen tubes grown in vitro with or without fusaoctaxin A. Scale bar represents 20 µm. Pollen tube length (bar graphs, left axis) and percentage of germinated pollen (lines, right axis) are charted. Data are means ± s.e.m. (n = 3). ns, no significant difference in student's *t*-test. **b,** The addition of fusaoctaxin A caused cell death of *N. benthamiana* leaves. Circles with broken line indicate infiltrated areas with indicated compounds. The experiment was replicated three times. GIAVSTAG: a control octapeptide. **c,** Inhibition effects of fusaoctaxin A on growth of mammalian cells were negligible. Positive Control is the inhibition of proliferation of BEL-7402 cells with Doxorubicin. The x-axis indicates the concentration of compounds and the y-axis represents the percentage of inhibition. FA: Fusaoctaxin A, M1: GIAVSTAG. Data are means ± s.d. (technical repeats n=3)

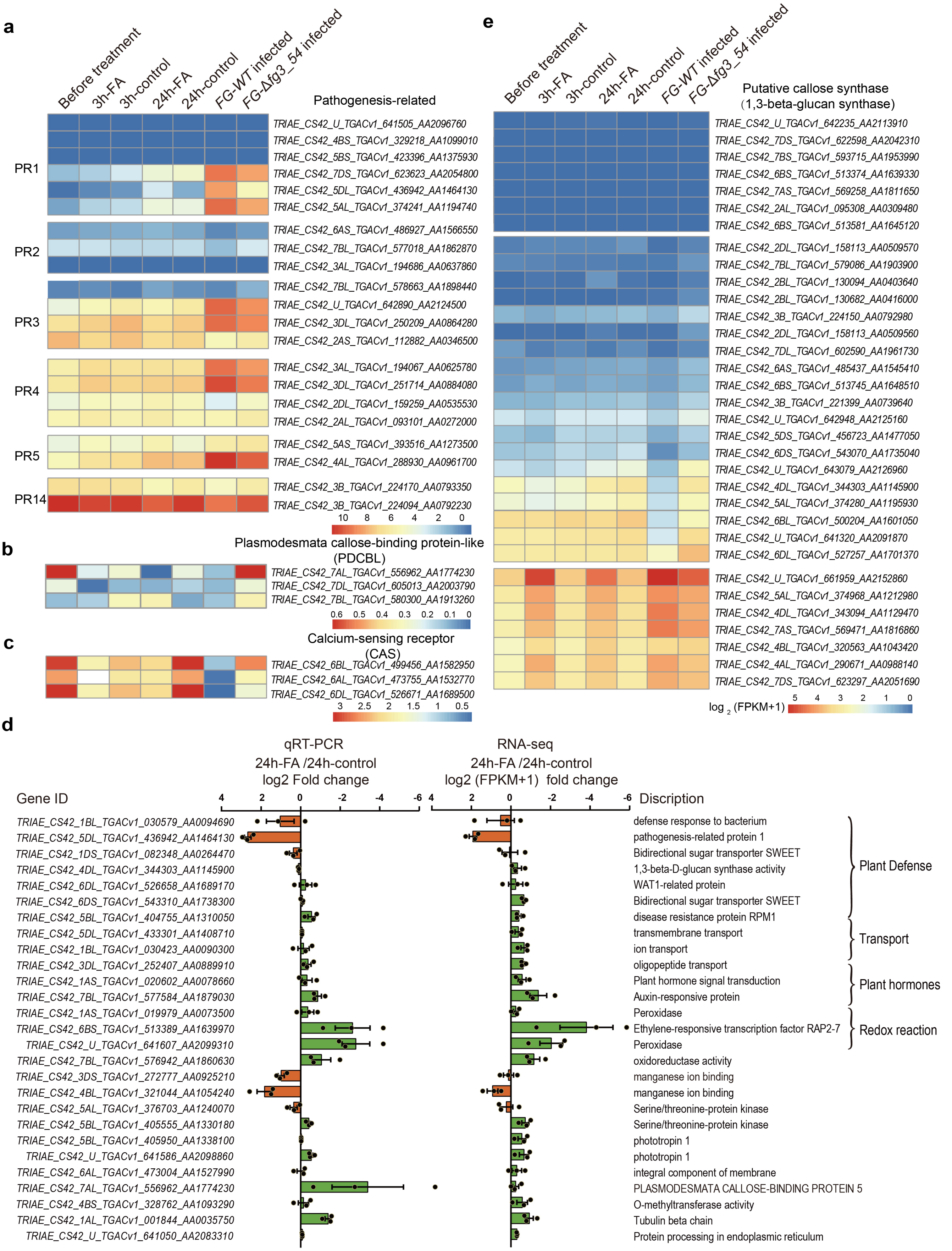

Supplementary Figure 10: Quantitative RT-PCR and RNA-seq analysis of wheat genes expression. a-c, Heat map showing the expression of wheat pathogenesis-related genes, plasmodesmata callose-binding protein-like genes and putative calcium-sensing receptor genes, based on the RNA-seq data. **d,** Charts showing the comparison of Quantitative RT-PCR (left) and RNA-seq results (right). Value represent the $\log_2$ (fold change) between wheat coleoptiles treated with fusaoctaxin A and control for 24 h. Data are means ± s.e.m. (n=3). Green represents down-regulated genes, and red represents up-regulated genes. **e,** Heat map showing the expression of wheat putative 1,3-beta-glucan

a

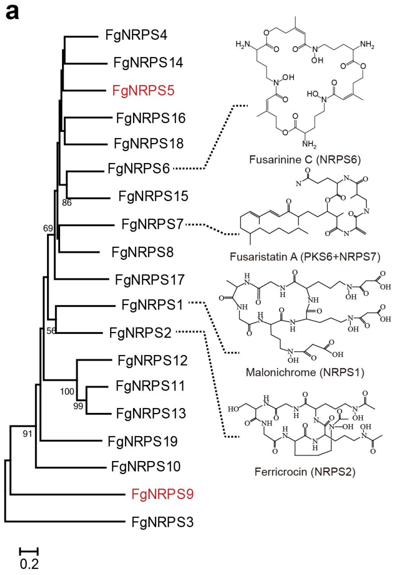

b

NRPS	A domain number
FgNRPS1	3
FgNRPS2	3
FgNRPS3	1
FgNRPS4	5
FgNRPS5	8
FgNRPS6	1
FgNRPS7	3
FgNRPS8	7
FgNRPS9	1
FgNRPS10	1
FgNRPS11	1
FgNRPS12	1
FgNRPS13	1
FgNRPS14	2
FgNRPS15	2
FgNRPS16	1
FgNRPS17	1
FgNRPS18	7
FgNRPS19	1
total	50

c **d**

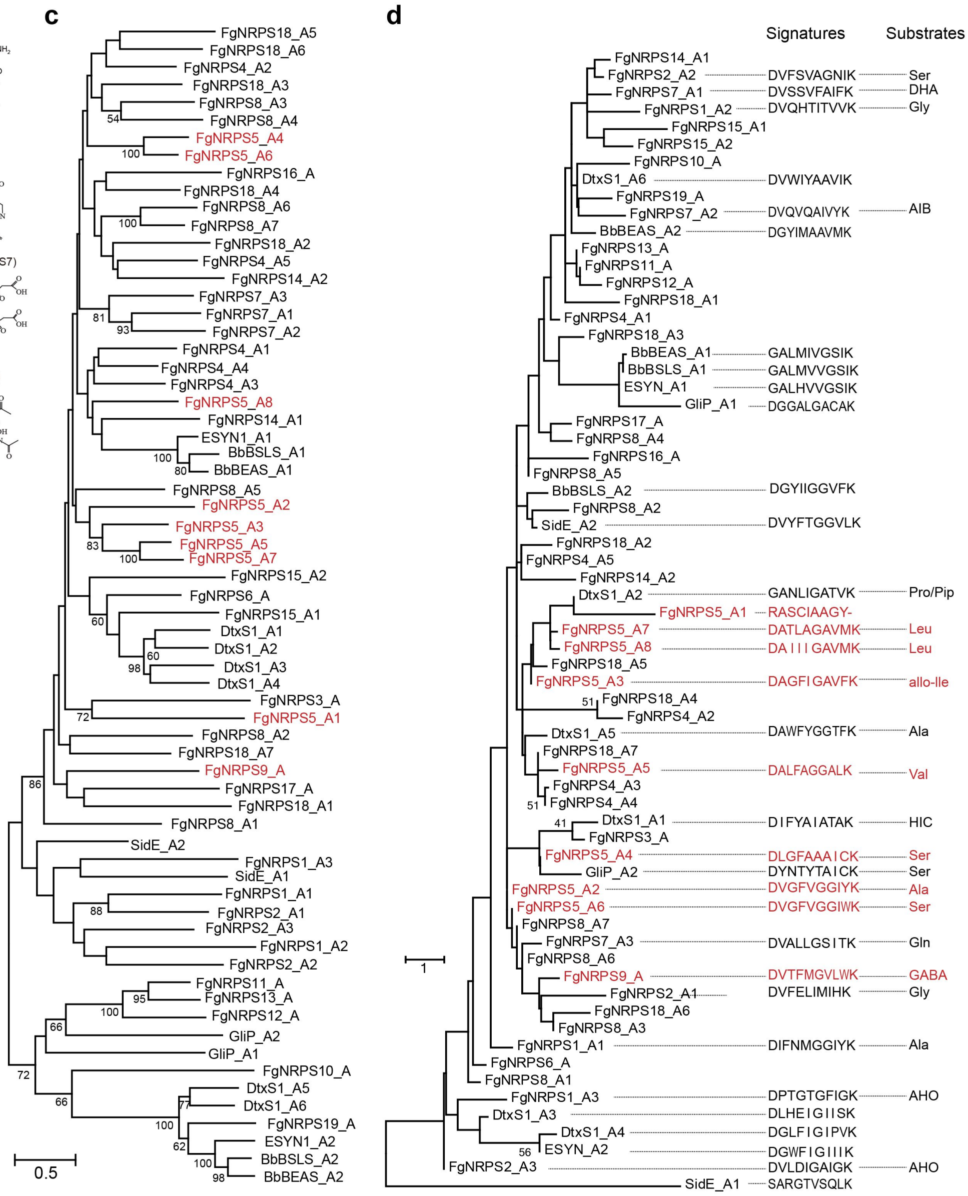

Supplementary Figure 11: Phylogenetic analysis of *F. graminearum* NRPS A domains and FgNRPS5 Stachelhaus codes. a, Phylogenetic tree obtained by Neighbor-Joining analysis of protein sequences of *F. graminearum* NRPSs. The numbers at the internal nodes indicate the percentage of replicate trees in which the associated taxa clustered together in a bootstrap test of 1,000 replicates. Four *F. graminearum* NRPS products are listed on the right[4]. **b,** A domain number of *F. graminearum* NRPSs. **c,** Maximum likelihood (ML) tree based on A domain protein sequences of F. graminearum and several other fungal NRPSs (LG model, gamma distributed, bootstrap=1,000). ESYN1, Enniatin synthetase of *F. equiseti*[5]; BbBSLS, Bassianolide synthetase of *Beauveria bassiana*[6]; BbBEAS, Beauvericin synthetase of *B. bassiana*[7]; SidE, Fumarylalanine synthetase of *Aspergillus fumigatus*[8]; Glip, Gliotoxin synthetase of *A. fumigatus*[9]; DtxS1, Dextruxin synthetase of *Metarhizium robertsii*[10]. d, ML tree based on A domain signatures (i.e. Stachelhaus code) (LG model, Gamma distributed, bootstrap=1,000). AHO, N-Hydroxy-ornithine; AIB, β-aminoisobutyric acid; DHA, dehydroalanine; HIC, α-hydroxyisocaproic acid; Pip, Pipecolic acid. NRPS protein sequences were analyzed with CDD[11] (conserved domains database) and NRPSpredictor2[12].

Supplementary Table 1: Plant materials and fungal strains used in this study.

Plant	Botanical name	Cultivar	Source
Wheat	*Triticum aestivum*	Zhongyuan 98-68	Guoshenmai 2004006, Henan, China
Wheat	*Triticum aestivum*	Bobwhite	CIMMYT Vasil, Vimla, et al. 1993[13]
Maize	*Zea mays*	B73	Russell, W. A.1972[14]; Schnable, P. S. et al. 2009[15]

Short name	Strain name	Identifier	Origin/Reference
FG-WT	*Fusarium graminearum* PH-1	Agricultural Research Collection NRRL 31084	Lab stock
Foc TR4	*Fusarium oxysporum* f.sp.cubense tropical race 4	Agricultural Research Collection NRRL 54006	Gift from Drs. Sijun Zheng[16]
F. avenaceum	*Fusarium avenaceum* M130-N458	China General Microbiological Culture Collection Center 3.3630	China General Microbiological Culture Collection Center
F. poae	*Fusarium poae* NF732	China General Microbiological Culture Collection Center 3.4601	China General Microbiological Culture Collection Center
V. dahliae	*Verticillium dahliae* V592	V592 from cotton originated in Xinjiang, China	Gift from Drs. Huishan Guo (Jin et al., 2018)[17]
M. robertsii	*Metarhizium robertsii*	Agricultural Research Service Collection of Entomopathogenic Fungal Cultures ARSEF 2575	Gift from Drs. Chengshu Wang

Strains	Generation method	Relevant characteristics	Source/Reference
FG-WT-AmCyan	pZD101	pvma3-AmCyan transformant of PH-1	Yuan et al., 2008[18]
FG-WT-RFP	JM135D	pEF1-RFP transformant of PH-1	This study
FG-ΔFGSG_13879	Split-marker PCR	*FGSG_13879* deletion mutant of PH-1	This study
FG-Δnrps5	Split-marker PCR	*nrps5* deletion mutant of PH-1	This study
FG-Δfgm9	Split-marker PCR	*fgm9* deletion mutant of PH-1	This study
FG-Δnrps9	Split-marker PCR	*nrps9* deletion mutant of PH-1	Zhang et al., 2012[2]
FG-Δfgm1	Split-marker PCR	*fgm1* deletion mutant of PH-1	This study
FG-Δfgm2	Split-marker PCR	*fgm2* deletion mutant of PH-1	Zhang et al., 2012[2]
FG-Δfgm3	Split-marker PCR	*fgm3* deletion mutant of PH-1	This study
FG-Δfgm4	Split-marker PCR	*fgm4* deletion mutant of PH-1	This study
FG-Δfgm4::fgm4	Split-marker PCR	*FG-* *Δfgm4* complemented transformant	This study
FG-Δfgm5	Split-marker PCR	*fgm5* deletion mutant of PH-1	Zhang et al., 2012[2]
FG-Δfg3_54	Split-marker PCR	*fg3_54* whole cluster deletion mutant of PH-1	This study
FG-ΔFGSG_10996	Split-marker PCR	*FGSG_10996* deletion mutant of PH-1	This study
FG-Δfg3_54-RFP	JM135D	pEF1-RFP transformant of *FG-Δfg3_54*	This study
FG-Δfg3_54-Amcyan	Flu6	pvma3-AmCyan transformant of *FG-Δfg3_54*	This study
FG-Δfgm4-RFP	JM135D	pEF1-RFP transformant of *FG-Δfgm4*	This study
FG-Δfgm5-Amcyan	Flu6	pvma3-AmCyan transformant of Δ*fgm5*	This study
FG-WT-OE::fgm4	WT-	pEF1-*FGM4* CDS transformant of PH-1	This study
FG-Δnrps5::fgm4	JM109A	pEF1-*FGM4* CDS transformant of *FG*-Δnrps5	This study
FG-Δfgm9::fgm4	JM109A	pEF1-*FGM4* CDS transformant of *FG*-Δ*fgm9*	This study
FG-Δnrps9::fgm4	JM109A	pEF1-*FGM4* CDS transformant of *FG* - Δ*nrps9*	This study
FG-Δfgm1::fgm4	JM109A	pEF1-*FGM4* CDS transformant of *FG*-Δ*fgm1*	This study
FG-Δfgm2::fgm4	JM109A	pEF1-*FGM4* CDS transformant of *FG*-Δ*fgm2*	This study
FG-Δfgm3::fgm4	JM109A	pEF1-*FGM4* CDS transformant of *FG-* Δ*fgm3*	This study
FG-Δfgm5::fgm4	JM109A	pEF1-*FGM4* CDS transformant of *FG-* Δ*fgm5*	This study
FG-Δfg3_54::fgm4	JM109A	pEF1-*FGM4* CDS transformant of *FG*-Δ*fg3_54*	This study
FGM4-RFP	JM65E	pEF1-*FGM4*-mRFP transformant of PH-1	This study
fgm4-eGFP	JM93	pvma3-eGFP-*FGM4* transformant of PH-1	This study
Foc TR4-eGFP		pvma3-eGFP transformant of Foc TR4	Gift from Prof. Sijun Zheng, South China Agricultural University, College of Natural Resources and Environment. (Zhang et al., 2018)[16]

Supplementary Table 2: Primers and plasmids used in this study.

A. Primers used in mutants generation and verification

Gene ID	Primer type	Primer sequence	Product length (bp)
FGSG_13879	Up-F	CGATGAGGAGAGAACGGAC	1004
	Up-R	TAGCCACGATTCGAAGCCGCGAGGGTGGATCTTATTGAC	
	Down-F	CGCATTGAATTGAAAAAGGAAGAGTATGGTCTGCGGATTGAGCCTATTG	976
	Down-R	GCATTGTCGGAGAGCGTG	
	IF	CACCCCATCATTACACCCGA	554
	IR	CAGAGCCCACCCGATACGTT	
	U	GTCAATAAGATCCCACCCTC	1854
	D	CAATAGGCTCAATCCGCAG	
FGSG_13878 *nrps5*	Up-F	CGGCACCTATTGACAACCAG	1182
	Up-R	TAGCCACGATTCGAAGCCGCCTCTGGGCGAGATTATGACA	
	Down-F	CGCATTGAATTGAAAAAGGAAGAGTATGGGTAATGATGTGTGTGTTGTGTG	1294
	Down-R	CCAGTGACATTTGTCGCAAG	
	IF	GCTCCTCTTCCGACACGGTG	651
	IR	ACATGATTGCCCAGCGTGCT	
	U	TGTCATAATCTCGCCCAGAG	4203
	D	CACACAACACACACATCATTACC	
FGSG_10989 *fgm9*	Up-F	TGAACGATGAAGCGAAAGGA	916
	Up-R	TAGCCACGATTCGAAGCCGCGGGTATTACAAGGATAAGTGATGC	
	Down-F	CGCATTGAATTGAAAAAGGAAGAGTATGCACGCCCAGGATAGTGTTAA	983
	Down-R	GGGTGATATGCAATGTCAGC	
	IF	CTCATCACCGGAGCAAGTCG	342
	IR	GGCGAGCAGGAATGTACCAG	
	U	GCATCACTTATCCTTGTAATACCC	1151
	D	TTAACACTATCCTGGGCGTG	
FGSG_10991 *fgm1*	Up-F	ACCATCAGTCTCTTGGAGCG	1012
	Up-R	TAGCCACGATTCGAAGCCGCGTTGGCGATTGGAAGTTATTG	
	Down-F	CGCATTGAATTGAAAAAGGAAGAGTATGGGCTCAAGTGCAGACTTCGT	1028
	Down-R	TGCTTTCGTCGAGTTATCGA	
	IF	TGTCCCAGGCGTAGTGGTTA	540
	IR	CCTCCTCTGGCACTGTTCTT	
	U	CAATAACTTCCAATCGCCAAC	1615
	D	ACGAAGTCTGCACTTGAGCC	
FGSG_10992 *fgm2*	Up-F	GTTGATGTTGTGTTGCTGGG	836
	Up-R	TAGCCACGATTCGAAGCCGCGGTCGTGCGATGATTTGAAA	
	Down-F	CGCATTGAATTGAAAAAGGAAGAGTATGAAATGTATTCAAGACTGCCAGAT	998
	Down-R	ATCATCGCAGCCAACACTCT	
	IF	GCCCACGATTCCAAACTCCA	528
	IR	ATGTCCGCTCAAGGACGTGTC	
	U	TTTCAAATCATCGCACGACC	1152
	D	ATCTGGCAGTCTTGAATACATTT	
FGSG_10993 *fgm3*	Up-F	GGTCGTGCGATGATTTGAAA	836
	Up-R	TAGCCACGATTCGAAGCCGCGTTGATGTTGTGTTGCTGGG	
	Down-F	CGCATTGAATTGAAAAAGGAAGAGTATGGGAGAGACTCTTTGTCGTTTTG	1013
	Down-R	TCAGCATGTAGTCCACGGAG	
	IF	CAATCAAGTGGTGGACACCG	525
	IR	CGTCTGCTTGGGATTCTCAC	
	U	CCCAGCAACACAACATCAAC	1480
	D	CAAAACGACAAAGAGTCTCTCC	
FGSG_10994 *fgm4*	Up-F	CATCCACGCTTTCATCATTG	1030
	Up-R	TAGCCACGATTCGAAGCCGCGATTGATTGGGTTACATACATCG	
	Down-F	CGCATTGAATTGAAAAAGGAAGAGTATGCTGCAAGGCATTGATTCACA	988
	Down-R	ATCCTGCTTTTAGCGAGGG	
	IF	GCAAGGCCAGATGATGTATCAA	867
	IR	GCCACACAACGTCAGCGTCT	
	U	CGATGTATGTAACCCAATCAATC	1403
	D	TGTGAATCAATGCCTTGCAG	
	FGM4 probe	ATGAATGGCTCTGCTGAACC	340
		CGCCGTAATTGTCCTTTGAA	
FG3_54	Up-F	TCTTCGCCATTCAGTCCAG	1030
	Up-R	TAGCCACGATTCGAAGCCGCACAGGAGGTTGAAGACATTGG	
	Down-F	CGCATTGAATTGAAAAAGGAAGAGTATGAGAAGGTCAGAAGGAAAGGTAGA	1081
	Down-R	TCGCCGATCATTTAACGAGT	
	IF-1	GCTCCTCTTCCGACACGGTG	651
	IR-1	ACATGATTGCCCAGCGTGCT	
	IF-2	GCAGGTCTCAAGCCAGTACG	532
	IR-2	TCCTGTGGCGAGTTGTAGCT	
FGSG_10996	Up-F	GATGTCGATGGAGGATTCAC	1154
	Up-R	TAGCCACGATTCGAAGCCGCTTTGAACGGTGAAGCCTAA	
	Down-F	CGCATTGAATTGAAAAAGGAAGAGTATGGAATTTCGCTTGCACTTTTG	1136
	Down-R	GCAATGGAGAAGAAGTAAGTG	
	IF	TGGTTGTCTTCCTGGTGATC	720
	IR	CTGTCGCCCTTGTAACCGTC	
	U	TTAGGCTTCACCGTTCAAA	2053
	D	CAAAAGTGCAAGCGAAATTC	
Hygromycin	HY	GATGTAGGAGGGCGTGGATATGTCCT	315
	YG	GTATTGACCGATTCCTTGCGGTCCGAA	
	Hyg-F	GCGGCTTCGAATCGTGGCTA	1596
	Hyg-R	CATACTCTTCCTTTTTCAATTCAATGCG	

B. Primers used in RT-PCR

Gene ID	Primer type	Primer sequence	Product length (bp)
tubulin	F	AGGTTGAGGACCAGATGCG	153
	R	CCTGGATAGAGGTGGAGTTTC	
FGSG_13878 *nrps5*	F	CCACTGAGCCACACGTCGGGTC	141
	R	GCCAGGGACATCGTGGCAGAAG	
FGSG_10989 *fgm9*	F (Supplementary Fig 7)	GTCCTTGGTGCTGCTGATGATGAGC	85
	R (Supplementary Fig 7)	CGGTAACGGCAACTGGAGGACTTCC	
	IF (Supplementary Fig 6)	CTCATCACCGGAGCAAGTCG	342
	IR (Supplementary Fig 6)	GGCGAGCAGGAATGTACCAG	
FGSG_10990 *nrps9*	F	CAACGACACGGGAAGTGGGC	417
	R	CAGGACTTTGGGACACCGCC	
FGSG_10991 *fgm1*	F	CTTGATGCCACACCCGACGAGATTC	81
	R	ACCTAGACACAGTCTTGCGCCGTAACC	
FGSG_10992 *fgm2*	F	GCCCACGATTCCAAACTCCA	528
	R	ATGTCCGCTCAAGGACGTGTC	
FGSG_10993 *fgm3*	F	TTCCTCCACTGTATTATAGT	375
	R	GCAGCTAGCTACGAGTTGAC	
FGSG_10994 *fgm4*	F	TGACATCGCCAATGACACGACACG	248
	R	ATCGGCCCCTTTCTCGAGTA	
FGSG_10995 *fgm5*	F	GGTCAATCCTTCGCCGTCGTCTT	170
	R	CGAGCCATGATCGGTTGCGAAT	
FgLaeA	F	ATTTGAGAAGTGGGCAGAGC	381
	R	TGCCCTGAATTTGTGTCCTG	
FGSG_10980	F	CGTTCGTATGGCTCAAGTCC	426
	R	CGACCAAGAAGCATCTGGAA	
TRIAE_CS42_1AL_TGACv1_001844_AA003575	F	GTACGAGGAAGAGGAAGAC	192
	R	CCTACATACGAACGCAGTA	
TRIAE_CS42_1AS_TGACv1_019979_AA007350	F	GAAATGTCCCTTCTTCTCTG	170
	R	GTTCCTCTTATCTTATGGTATCG	
TRIAE_CS42_1AS_TGACv1_020602_AA007866	F	CAAGGAAGTAAGAAGATAGCATA	157
	R	TCACACAACAAAGAACTAGG	
TRIAE_CS42_1BL_TGACv1_030579_AA009469	F	CAAGTGCCTCCTGGTGAC	167
	R	ACTGGTAGTTGACGGTGAG	
TRIAE_CS42_4DL_TGACv1_344303_AA114590	F	TTCCACTCTGTTCTACCTAC	102
	R	TCTTCCTTCAGTTCTTCATTATC	
TRIAE_CS42_5BL_TGACv1_404755_AA131005	F	AGACTTGGAGTGGCATAC	200
	R	AGAGCATTCAGCAGAGATAA	
TRIAE_CS42_5BL_TGACv1_405950_AA133810	F	CGCAATGTAGTCCTGTATC	159
	R	TCCTCCTGGTAGTTGTTG	
TRIAE_CS42_5DL_TGACv1_433301_AA140871	F	TCTTCTGAGCACCAACTAA	100
	R	ATGGCTTCCTCTGTCAAT	
TRIAE_CS42_7AL_TGACv1_556962_AA177423	F	CGTGTTTCCTTCCAGTTC	117
	R	AATGACCAGGTGTTGAGT	
TRIAE_CS42_U_TGACv1_641050_AA2083310	F	TTATACCATAGGCACACTCAA	105
	R	AGTCATATCTCATATCTCAGTTGT	
TRIAE_CS42_U_TGACv1_641586_AA2098860	F	GTGGATGGACAGACAGAG	100
	R	CCTAAGTACGAGTACGAACA	
TRIAE_CS42_1BL_TGACv1_030423_AA009030	F	CACGCTCATAGTTACCAT	106
	R	CAGCCAGTAGAAGTAGTC	
TRIAE_CS42_3DL_TGACv1_252407_AA088991	F	GATGACACCTACGCTGCTA	157
	R	CGGATGAGACGAAGAGATGA	
TRIAE_CS42_4BS_TGACv1_328762_AA109329	F	GACACCGTTCTTCTCAAGT	135
	R	TCCGACCACCATATCCAT	
TRIAE_CS42_6AL_TGACv1_473004_AA152799	F	ACATCCTTGGCTTCACCTT	101
	R	CTCCTTCACCTCCTTCACAT	
TRIAE_CS42_7BL_TGACv1_576942_AA186063	F	CTGTCGCTTGATGCTAATGT	200
	R	CTCTTCCGCCACTTGTTG	
TRIAE_CS42_3DS_TGACv1_272777_AA092521	F	AAGGAAGGGTGATGTGTT	134
	R	CAGCGTTAGCAATGGTAAT	
TRIAE_CS42_4BL_TGACv1_321044_AA105424	F	GTTGATTACACGGAAGATTAGT	112
	R	GGCTTACAGACATACAATGG	
TRIAE_CS42_5DL_TGACv1_436942_AA146413	F	GCATAAAGAATAAAGCCGAGAT	110
	R	CCTACAATGATAGTACAACTTACA	
TRIAE_CS42_1DS_TGACv1_082348_AA026447	F	AACAAGCGATGGAGAATG	156
	R	GATGGATACACTAATGAATACGA	
TRIAE_CS42_5AL_TGACv1_376703_AA124007	F	TAGGAGTTGCTAGAGGATTG	168
	R	TTGTCAGGATTCGGCTAA	
TRIAE_CS42_5BL_TGACv1_405555_AA133018	F	CCGATGCTGAGACTACTT	157
	R	TTCTTCCTTCATTCCTTCCA	
TRIAE_CS42_6BS_TGACv1_513389_AA163997	F	CATCCATCTTCGCATCATAT	155
	R	GCTGGTTGGTTACATATCAT	
TRIAE_CS42_6DL_TGACv1_526658_AA168917	F	GGTGCTGATGGTACTGTA	184
	R	GTTGGACTGGAGGATGAA	
TRIAE_CS42_6DS_TGACv1_543310_AA173830	F	CTCAACACAACCGTCAAC	166
	R	TATGGACTAACTCACTGGATG	
TRIAE_CS42_7BL_TGACv1_577584_AA187903	F	GGATGCTTGTTGGTGATG	124
	R	TTCTGGTTCTTGCTCTTCT	
TRIAE_CS42_U_TGACv1_641607_AA2099310	F	TATCCGTCCGTCCTAAGT	158
	R	CATAGTGGTTCGCTGGTA	

C. Plasmids used in this study

Use	Plasmid name	Relevant characteristics	Plasmid backbone	Primers	Template	Cloning strategy
Generation fluorescence tagged *F. graminearum* strains	pZD101	pvma3-AmCyan, Hyg^R-A^R	Previously generated in Yuan et al., 2008[18]			
	Flu19	pEF1-insertion site-mRFP, Hyg^R-A^R	pZD101	PC1/PC2	Genomic DNA of PH-1	HindIII - Spe I
	Flu6	pvma3-AmCyan, Neo^R-A^R	pZD101			
	JM135D	pEF1-RFP, Neo^R-A^R	Flu6		Flu19	HindIII - EcoR I
fgm4 Overexpression	JM82	pMD19-FGM4 (cDNA, 834 bp), A^R	pMD19-T	PC3/PC4	cDNA of PH-1 infected wheat coleoptiles	
	JM82A	pMD19-FGM4 (cDNA), A^R	pMD19-T	PC5/PC6	JM82	Spe I - Nde I
	JM65	pEF1-FGM4, Hyg^R-A^R	pZD101		JM82	Spe I - Nde I
	JM109	pEF1-FGM4, Neo^R-A^R	JM135D		JM82	Spe I - Nde I
fgm4 complement	JM45	pMD19-Neo, A^R	pMD19-T	PC7/PC8	Flu6	EcoR I - Not I
	JM70	pMD19-Native pro-FGM4 (gDNA), Neo^R-A^R	JM45	PC9/PC10	Genomic DNA of PH-1	Pst I - Not I
Subcellular localization	JM65E	pEF1-FGM4-mRFP, Hyg^R-A^R	Flu19	PC5/PC11	JM82	Spe I - Nde I
	W33	ProLAT52:eGFP	Previously generated in Zhang et al., 2008[19]			
	JM89A	pMD19-eGFP (without TAA)	pMD19-T	PC12/PC13	W33	Spe I - Kpn I
	JM92	pvma3-eGFP, Hyg^R-A^R	pZD101		JM82	Spe I - Kpn I
	JM93	pvma3-eGFP-FGM4, Hyg^R-A^R	JM92		JM82	Kpn I - Nde I
FgLaeA OX	JM79A	pEF1-FgLaeA, Hyg^R-A^R	JM65	PC14/PC15	cDNA of PH-1	Spe I - Xho I
Yeast two-hybrid	JM80A	pMD19-FgLaeA (cDNA), A^R	pMD19-T	PC16/PC17	JM79A	BamH I - Not I
	JM95	BK-FgLaeA	pGBKT7		JM80A	BamH I - Not I
	JM82C	pMD19-FGM4 (cDNA), A^R	pMD19-T	PC3/PC18	JM82	EcoR I - BamH I
	JM97	AD-FGM4	pGADT7		JM82C	EcoR I - BamH I
	JM86	BK-FGM4	pGBKT7		JM82	EcoR I - Not I
	JM98	BK-FGM4 (1-245)	pGBKT7	PC3/PC19	JM82	EcoR I - Not I
	JM99	BK-FGM4 (1-212)	pGBKT7	PC3/PC20	JM82	EcoR I - Not I
Southern blot probes	JM57	*FGM9* probe （Spe I - EcoR I）	pMD19-T	*FGM9*-IF/*FGM9*-IR	Genomic DNA of PH-1	
	JM58	*FGM1* probe （Spe I - EcoR I）	pMD19-T	*FGM1*-IF/*FGM1*-IR	Genomic DNA of PH-1	
	JM59	*FGM3* probe （Nhe I - Kpn I）	pMD19-T	*FGM3*-IF/*FGM3*-IR	Genomic DNA of PH-1	
	JM60	*FGM4* probe （Spe I - EcoR V）	pMD19-T	PC21/PC22	Genomic DNA of PH-1	
	JM56	*FgNRPS5* probe （Spe I - EcoR I）	pMD19-T	PC23/PC24	Genomic DNA of PH-1	

D. Primers used for plasmids construction

Plasmids	Primer ID	Sequence	Product length (bp)	Enzyme sites
Flu19	PC1	CC**AAGCTT**GTGCAAGCATCCATGATTGA	1045	HindIII
	PC2	GG**ACTAGT**TTGACGGTTGTGGATGGTT		Spe I
JM82	PC3	GT**GAATTC**ATGGACTCGTTGCTTAATTCAA	842	EcoR I
	PC4	TCAGCAAACTTGAGCTCCAT		
JM82A	PC5	GG**ACTAGT**ATGGACTCGTTGCTTAATTCAA	848	Spe I
	PC6	**CATATG**TCAGCAAACTTGAGCTCCAT		Nde I
JM45	PC7	CG**GAATTC**CGACGAATAGGAACTTCGGA	2113	EcoR I
	PC8	TT**GCGGCCGC**ATTTCCATTCGCCATTCAGG		Not I
JM70	PC9	AA**CTGCAG**AGTGCAACCTGACACAGCCAT	2401	Pst I
	PC10	TT**GCGGCCGC**ACCAACGGAAATCGGCATTT		Not I
JM65E	PC5	see above	847	Spe I
	PC11	CC**CATATG**GCAAACTTGAGCTCCATGTT		Nde I
JM89A	PC12	CC**ACTAGT**ATGGTGAGCAAGGGCGAGGA	733	Spe I
	PC13	GT**GGTACC**CTTGTACAGCTCGTCCATGCCG		Kpn I
JM79A	PC14	G**ACTAGT**ATGGCTGTGATGCCTCCACCA	967	Spe I
	PC15	CCG**CTCGAG**TTACTGTGGCGGGCCAGGC		Xho I
JM80A	PC16	G**GGATCC**TCATGGCTGTGATGCCTCCACCA	969	BamH I
	PC17	G**GCGGCCGC**TTACTGTGGCGGGCCAGGC		Not I
JM82C	PC3	see above	849	EcoR I
	PC18	G**GGATCC**TCAGCAAACTTGAGCTCCAT		BamH I
JM98	PC3	see above	756	EcoR I
	PC19	TT**GCGGCCGC**TTAGGAATATACATCGGCCCCTT		Not I
JM99	PC3	see above	657	EcoR I
	PC20	TT**GCGGCCGC**TTACTCGTTGACGTTGGCACCGT		Not I
JM60	PC21	GCCTGCTATGATTCCAACCT	558	
	PC22	ACACGACACGTTATGCCATC		
JM56	PC23	TGCATAGCGCAGAGCAAGTG	390	
	PC24	ACCGGCACCATGACACAGAC		

Supplementary Table 3: RNA-seq expression data of *F. graminearum* secondary metabolic genes from *fgm4* overexpressing strains growing in YEPD for 3 days.

A. Expression data of *fg3_54* and flanking genes

		Average FPKM value				Average Reads count			
FGSG ID	Gene	WT	Δ*fgm4*	WT-OE::*FGM4*	Δ*fg3_54*-OE::*FGM4*	WT	Δ*fgm4*	WT-OE::*FGM4*	Δ*fg3_54*-OE::*FGM4*
FGSG_11000		0.0	0.0	0.2	0.1	0	0	1	0
FGSG_10999		0.0	0.0	0.0	0.0	0	0	0	0
FGSG_10998		11.8	4.1	3.2	3.2	226	79	62	62
FGSG_10997		3.1	2.1	6.4	3.3	37	24	74	37
FGSG_10996		21.0	37.7	35.2	47.2	603	1069	987	1341
FGSG_10995	*fgm5*	1.1	49.2	21.9	0.0	66	2975	1356	0
FGSG_10994	*fgm4*	2.5	0.0	1452.3	735.5	22	0	12888	6474
FGSG_10993	*fgm3*	34.1	173.7	3038.3	0.2	569	2880	47897	3
FGSG_10992	*fgm2*	9.3	56.9	675.0	0.0	124	732	8360	0
FGSG_10991	*fgm1*	0.6	10.5	1369.6	0.1	12	215	27232	2
FGSG_10990	*nrps9*	0.7	0.6	1905.4	0.1	25	21	64871	3
FGSG_10989	*fgm9*	1.2	0.5	585.5	0.1	14	6	6930	2
FGSG_13878	*nrps5*	0.2	0.1	25.2	0.0	113	54	12883	0
FGSG_13879		0.0	0.0	0.0	0.0	1	0	1	0
FGSG_10987		0.2	0.3	0.6	0.7	3	4	10	11
FGSG_10986		0.6	4.6	1.7	9.5	15	118	42	237
FGSG_10985		0.7	1.5	1.3	1.3	8	18	14	15
FGSG_10984		0.2	0.5	0.4	0.2	4	10	10	5
FGSG_10983		12.5	11.7	33.1	16.2	184	168	472	231
FGSG_10982		2.5	1.4	1.7	1.9	78	45	54	59
FGSG_13880		65.8	120.1	181.2	157.0	747	1330	1965	1732
FGSG_13881		5.5	6.0	9.7	9.4	124	131	209	201
FGSG_10980		2.2	3.1	2.9	2.1	41	56	51	37

B. Expression data of *F. graminearum PKS* s and *NRPS* s

		Average FPKM value				Average Reads count			
FGSG ID	Gene	WT	Δ*fgm4*	WT-OE::*FGM4*	Δ*fg3_54*-OE::*FGM*	WT	Δ*fgm4*	WT-OE::*FGM4*	Δ*fg3_54*-OE::*FGM*
FGSG_10548	*FgPKS1*	0.0	0.0	0.0	0.0	5	5	6	3
FGSG_04694	*FgPKS2*	0.3	0.1	0.1	0.0	34	9	13	5
FGSG_09182	*FgPKS3*	0.1	0.3	0.3	0.3	9	24	29	24
FGSG_12126	*FgPKS4*	0.1	0.1	0.2	0.1	7	7	18	6
FGSG_05794	*FgPKS5*	0.2	0.2	0.2	0.2	29	25	28	28
FGSG_08208	*FgPKS6*	0.1	0.2	0.5	0.1	8	20	57	16
FGSG_08795	*FgPKS7*	1.8	1.7	1.1	1.2	194	177	117	126
FGSG_03340	*FgPKS8*	3.5	1.8	5.0	1.6	402	213	573	189
FGSG_10464	*FgPKS9*	0.1	0.2	0.6	0.4	12	21	67	44
FGSG_07798	*FgPKS10*	0.2	0.3	2.2	0.2	39	44	386	27
FGSG_01790	*FgPKS11*	0.0	0.0	0.0	0.0	0	1	0	0
FGSG_02324	*FgPKS12*	0.2	0.1	0.1	0.1	16	5	8	9
FGSG_02395	*FgPKS13*	0.0	0.0	0.0	0.0	1	1	1	1
FGSG_03964	*FgPKS14*	0.1	0.0	0.0	0.0	10	3	3	1
FGSG_04588	*FgPKS15*	0.0	0.1	3.3	1.0	2	8	324	95
FGSG_11026	*FgNRPS1*	0.0	0.0	0.0	0.0	1	0	1	2
FGSG_05372	*FgNRPS2*	7.5	5.6	7.9	3.5	1733	1253	1756	774
FGSG_10523	*FgNRPS3*	2.2	1.3	2.3	1.2	256	146	265	136
FGSG_02315	*FgNRPS4*	22.0	8.5	6.5	9.0	7746	2973	2283	3151
FGSG_13878	*FgNRPS5*	0.2	0.1	25.2	0.0	113	54	12883	0
FSGS_03747	*FgNRPS6*	2.4	1.2	2.7	1.7	222	110	249	155
FGSG_08209	*FgNRPS7*	0.5	2.6	20.6	2.4	109	523	4186	475
FGSG_11659	*FgNRPS8*	0.7	1.0	0.3	0.2	142	213	52	35
FGSG_10990	*FgNRPS9*	0.7	0.6	1905.4	0.1	25	21	64871	3
FGSG_06507	*FgNRPS10*	10.1	10.8	5.7	7.7	597	637	336	457
FGSG_03245	*FgNRPS11*	0.1	0.3	0.1	0.2	3	12	6	8
FGSG_11294	*FgNRPS12*	0.1	0.0	0.1	0.0	5	2	3	1
FGSG_13153	*FgNRPS13*	0.5	0.1	0.4	0.1	22	5	17	5
FGSG_11395	*FgNRPS14*	0.0	0.0	0.0	0.0	1	1	2	0
FGSG_02394	*FgNRPS15*	0.0	0.0	0.0	0.0	0	1	0	0
FGSG_01680	*FgNRPS16*	0.1	0.0	0.1	0.2	8	4	7	11
FGSG_10702	*FgNRPS17*	0.0	0.0	0.0	0.0	0	0	0	0
FGSG_13783	*FgNRPS18*	0.0	0.0	0.0	0.0	0	0	0	0
FGSG_11989	*FgNRPS19*	0.0	0.2	0.2	0.1	1	10	9	7
FGSG_03066	*DTC1*	14.1	32.8	5.5	7.3	332	756	126	163
FGSG_07673	*STC1*	768.0	1518.0	1628.1	976.6	3375	6688	6725	4201
FGSG_00451	*STC2*	1.1	1.5	1.7	1.3	11	14	16	13
FGSG_03494	*STC4*	10.1	5.3	13.4	8.7	136	69	172	113
FGSG_08181	*STC5*	0.0	0.0	0.0	0.0	0	0	1	0
FGSG_11327	*STC6*	0.0	0.0	0.2	0.0	0	0	2	0
FGSG_10397	*STC9*	0.0	0.0	0.0	0.0	0	0	1	0

C. Expression data of TRI cluster involved in DON production

		Average FPKM value				Average Reads count			
FGSG NO.	Gene	WT	Δ*fgm4*	WT-OE::*FGM4*	Δ*fg3_54*-OE::*FGM*	WT	Δ*fgm4*	WT-OE::*FGM4*	Δ*fg3_54*-OE::*FGM*
FGSG_00070	*TRI16*	0.3	0.5	0.8	0.8	6	9	16	15
FGSG_00071	*TRI1*	3.0	1.9	2.2	2.3	76	48	54	60
FGSG_03532	*TRI8*	3.9	2.2	2.4	2.8	66	36	40	46
FGSG_03533	*TRI7*	0.2	0.1	0.0	0.1	1	0	0	0
FGSG_03534	*TRI3*	0.0	0.0	0.1	0.1	0	0	1	2
FGSG_03535	*TRI4*	0.0	0.2	0.0	0.3	0	4	0	7
FGSG_03536	*TRI6*	0.5	0.3	0.2	0.2	4	2	2	2
FGSG_03537	*TRI5*	0.0	0.0	0.1	0.3	1	0	1	5
FGSG_03538	*TRI10*	1.5	0.6	0.3	0.6	25	10	4	10
FGSG_03539	*TRI9*	1.3	0.7	0.9	0.7	8	4	5	4
FGSG_03540	*TRI11*	0.1	0.0	0.0	0.1	1	0	0	1
FGSG_03541	*TRI12*	1.0	0.8	1.3	0.9	24	18	31	22
FGSG_03542	*TRI13*	2.5	0.0	0.2	0.2	4	0	0	0
FGSG_03543	*TRI14*	0.6	0.2	0.4	0.3	9	3	5	5
FGSG_07896	*TRI101*	37.4	41.5	25.6	50.7	709	802	487	980

Supplementary Table 4: Accession numbers

Gene	Species	Gene NO.	Annotation	GenBank accession NO.	
		FGSG_13879	Probable tyrocidine synthetase	XM_011327081.1	
Fgnrps5		FGSG_13878 (FGSG_17487)	Related to non-ribosomal peptide synthetase	XM_011327080.1	KY111279
fgm9		FGSG_10989	Conserved hypothetical protein	XM_011327079.1	
Fgnrps9		FGSG_10990	Related to AM-toxin synthetase (AMT)	XM_011327078.1	
fgm1		FGSG_10991	Related to benzoate 4-monooxygenase cytochrome P450	XM_011327077.1	
fgm2		FGSG_10992	Related to polysaccharide deacetylase	XM_011327076.1	
fgm3		FGSG_10993	Related to selenocysteine lyase	XM_011327075.1	
fgm4		FGSG_10994	Conserved hypothetical protein	XM_011327074.1	KY111280
fgm5		FGSG_10995	Related to multidrug resistance protein	XM_011327073.1	
		FGSG_10996	Conserved hypothetical protein	XM_011327072.1	
Tubulin		FGSG_09530	Beta-tubulin	XM_011329885.1	
FgLaeA		FGSG_00657 (FGSG_15765)	Conserved hypothetical protein	XM_011318048.1	
RPB2	*F . fujikuroi* IMI 58289	FFUJ_07996	Probable DNA-dependent RNA polymerase II RPB140 (RPE	HF679027.1	
RPB2	*F. verticillioides* 7600	FVEG_09286	DNA-directed RNA polymerase II subunit RPB2	DS022253.1	
RPB2	*F. temperatum* CMWF389			LJGR01000004.1	
RPB2	*F. circinatum* strain GL1327			JRVE01000005.1	
RPB2	*F. agapanthi* NRRL 54464			LTDI01000291.1	
RPB2	*F. oxysporum* Fo5176	FOXB_01326	Hypothetical protein	AFQF01000478.1	
RPB2	*F. oxysporum* f. sp. conglutinans race 2	FOPG_06891	DNA-directed RNA polymerase II subunit RPB2	JH658835.1	
RPB2	*F. oxysporum* f. sp. vasinfectum 25433	FOTG_10105	DNA-directed RNA polymerase II subunit RPB2	JH657942.1	
RPB2	*F. oxysporum* f. sp. pisi HDV247	FOVG_06900	DNA-directed RNA polymerase II subunit RPB2	JH650971.1	
RPB2	*F. oxysporum* f. sp. raphani 54005	FOQG_05936	DNA-directed RNA polymerase II subunit RPB2	JH658371.1	
RPB2	*F. oxysporum* f. sp. cubense tropical race 4	FOIG_08965	DNA-directed RNA polymerase II subunit RPB2	JH658285.1	
RPB2	*F. oxysporum* FOSC 3-a	FOYG_08835	DNA-directed RNA polymerase II subunit RPB2	JH717843.1	
RPB2	*F. oxysporum* f. sp. melonis 26406	FOMG_07427	DNA-directed RNA polymerase II subunit RPB2	JH659332.1	
RPB2	*F. oxysporum* f. sp. lycopersici 4287	FOXG_10639	DNA-directed RNA polymerase II subunit RPB2	CM000595.1	
RPB2	*F. oxysporum* Fo47	FOZG_10131	DNA-directed RNA polymerase II subunit RPB2	JH717901.1	
RPB2	*F. oxysporum* f. sp. lycopersici MN25	FOWG_14904	DNA-directed RNA polymerase II subunit RPB2	JH650845.1	
RPB2	*F. oxysporum* f. sp. radicis-lycopersici 26381	FOCG_04994	DNA-directed RNA polymerase II subunit RPB2	JH651387.1	
RPB2	*F. avenaceum* Fa05001	FAVG1_00503	Hypothetical protein	JPYM01000001.1	
RPB2	*F. acuminatum* CS5907	BN851_0029680		CBMG010000552.1	
RPB2	*F. pseudograminearum* CS3096	FPSE_00366	Hypothetical protein	AFNW01000008.1	
RPB2	*F. culmorum* CS7071	BN852_0028830		CBMH010000367.1	
RPB2	*F. graminearum* PH-1	FGSG_02659	DNA-directed RNA polymerase	CM000574.1	
RPB2	*F. graminearum* CS3005	FG05_02659	Hypothetical protein	JATU01000019.1	
RPB2	*F. langsethiae* Fl201059	FLAG1_02775	DNA-directed RNA polymerase II subunit RPB2	JXCE01000028.1	
RPB2	*F. poae* 2516	FPOA_02651	Hypothetical protein	LYXU01000001.1	
RPB2	*F. sambucinum* F-4			LSRD01000041.1	
RPB2	*F. equiseti* CS3069	BN850_0013520		CBMI010000268.1	
RPB2	*F. solani* f. pisi 77-13-4	NECHADRAFT_70495	Hypothetical protein	GG698912.1	
RPB2	*M. anisopliae* ARSEF 23	MAA_00336	DNA-dependent RNA polymerase II beta subunit	ADNJ02000003.1	
RPB2	*M. acridum* CQMa 102	MAC_02463	DNA-dependent RNA polymerase II beta subunit	GL698481.1	
RPB2	*B. bassiana* ARSEF 2860	BBA_10291	DNA-dependent RNA polymerase II beta subunit	JH725267.1	
RPB2	*C. salicis* CBS 607.94	CSAL01_03721	DNA-directed RNA polymerase II subunit RPB2	JFFI01001504.1	
RPB2	*C. simmondsii* CBS122122	CSIM01_13490	DNA-directed RNA polymerase II subunit RPB2	JFBX01000673.1	
RPB2	*C. nymphaeae* SA-01	CNYM01_12501	DNA-directed RNA polymerase II subunit RPB2	JEMN01001610.1	
RPB2	*C. fioriniae* PJ7	CFIO01_11396	DNA-directed RNA polymerase II subunit RPB2	JARH01001071.1	
RPB2	*C. higginsianum* IMI 349063	CH063_00471	DNA-directed RNA polymerase II subunit RPB2	CACQ02006013.1	
RPB2	*C. tofieldiae* 0861 Ct v4	CT0861_11606	DNA-directed RNA polymerase II subunit RPB2	LFIV01000346.1	
RPB2	*C. graminicola* M1.001	GLRG_03562	RNA polymerase Rpb2	GG697340.1	
RPB2	*C. orbiculare* MAFF 240422	Cob_08568	DNA-directed RNA polymerase II subunit RPB2	KB725924.1	
RPB2	*V. dahliae* VdLs.17	VDAG_05625	DNA-directed RNA polymerase II subunit RPB2	DS572704.1	
RPB2	*N. crassa* OR74A	NCU05120	DNA-dependent RNA polymerase II RPB140	CM002241.1	
RPB2	*M. grisea* 70-15	MGG_04714	DNA-directed RNA polymerase II subunit RPB2	CM001231.1	
RPB2	*S. cerevisiae* S288c			BK006948.2	
b-ANK	*F. graminearum* CS3005	FG05_10994	Hypothetical protein	CM002653.1	
b-ANK	*F. culmorum* CS7071	BN852_0075430		CBMH010001032.1	
b-ANK	*F. pseudograminearum* CS3096	FPSE_02489	Hypothetical protein	AFNW01000059.1	
b-ANK	*F. oxysporum* FOSC 3-a	FOYG_10529	Hypothetical protein	AFML01000090.1	
b-ANK	*F. acuminatum* CS5907			CBMG010001915.1	
b-ANK	*F. avenaceum* Fa05001			JPYM01000019.1	
b-ANK	*F. langsethiae* Fl201059	FLAG1_11245	Hypothetical protein	JXCE01000791	
b-ANK	*C. tofieldiae* 0861	CT0861_10344	Ankyrin repeat-containing protein	LFIV01000182.1	
b-ANK	*C. simmondsii* CBS122122	CSIM01_03349	Hypothetical protein	JFBX01000347.1	
b-ANK	*C. nymphaeae* SA-01	CNYM01_01474	Hypothetical protein	JEMN01001464.1	
b-ANK	*C. fioriniae* PJ7	CFIO01_03411	Hypothetical protein	JARH01000413.1	
b-ANK	*C. salicis* CBS 607.94	CSAL01_09694	Hypothetical protein	JFFI01001557.1	
Apf2	*F . fujikuroi* IMI 58289	FFUJ_00012	Uncharacterized protein	CCT63352.1	
b-ANK	*F. langsethiae* Fl201059	FLAG1_08954	Hypothetical protein	JXCE01000303	
TOXE	*Cochliobolus carbonum* -AF038874	TOXE	*Cochliobolus carbonum* TOXE protein	AF038874.1	
b-ANK	*Bipolaris zeicola* 26-R-13	COCCADRAFT_113496	Hypothetical protein	KI965164.1	
		CSIM01_03354	Cytochrome P450		
		CSIM01_03353	RTA1 domain-containing protein		
		CSIM01_03352	3-isopropylmalate dehydrogenase		
		CSIM01_03351	Branched-chain-amino-acid aminotransferase		
		CSIM01_03350	3-isopropylmalate dehydratase large subunit 2		
CaFGM4		CSIM01_03349	Ankyrin repeat-containing protein		
CaFGM1		CSIM01_03348	Cytochrome P450		
CaFGM2	*C. simmondsii* CBS122122	CSIM01_03347	Peptidoglycan deacetylase	JFBX01000347.1	
CaFGM3		CSIM01_03346	Aspartate aminotransferase		
		CSIM01_03345	2-isopropylmalate synthase		
		CSIM01_03344	Major facilitator superfamily		
		CSIM01_03343	NRPS		
		CSIM01_03342	C6 zinc finger domain-containing protein		
		CSIM01_03341	Methylisocitrate lyase		
		CSIM01_03340	Fungal transcription factor regulatory middle homology regi		
		CSIM01_03339	Major facilitator superfamily		

Supplementary References:

1. Seong, K.-Y. et al. Conidial germination in the filamentous fungus *Fusarium graminearum*. *Fungal Genet. Biol.* 45, 389–399 (2008).
2. Zhang, X.-W. et al. In planta stage-specific fungal gene profiling elucidates the molecular strategies of *Fusarium graminearum* growing inside wheat coleoptiles. *Plant Cell* tpc–112 (2012).
3. Zhang, Y. et al. Cellular tracking and gene profiling of *Fusarium graminearum* during maize stalk rot disease development elucidates its strategies in confronting phosphorus limitation in the host apoplast. *PLoS Pathog.* 12, e1005485 (2016).
4. Hansen, F. T. et al. An update to polyketide synthase and non-ribosomal synthetase genes and nomenclature in *Fusarium*. *Fungal Genet. Biol.* 75, 20–29 (2015).
5. Haese, A. et al. Molecular characterization of the enniatin synthetase gene encoding a multifunctional enzyme catalysing N-methyldepsipeptide formation in *Fusarium scirpi*. *Mol. Microbiol.* 7, 905–914 (1993).
6. Xu, Y. et al. Biosynthesis of the cyclooligomer depsipeptide bassianolide, an insecticidal virulence factor of *Beauveria bassiana*. *Fungal Genet. Biol.* 46, 353–364 (2009).
7. Xu, Y. et al. Biosynthesis of the cyclooligomer depsipeptide beauvericin, a virulence factor of the entomopathogenic fungus *Beauveria bassiana*. *Chem. Biol.* 15, 898–907 (2008).
8. Steinchen, W. et al. The bimodular peptide synthetase SidE produces fumarylalanine in the human pathogen *Aspergillus fumigatus*. *Appl. Environ. Microbiol.* AEM–02642 (2013).
9. Cramer, R. A. et al. Disruption of a nonribosomal peptide synthetase in *Aspergillus fumigatus* eliminates gliotoxin production. *Eukaryot. Cell* 5, 972–980 (2006).

10.Wang, B. et al. Unveiling the biosynthetic puzzle of destruxins in *Metarhizium* species. *Proc. Natl. Acad. Sci.* 109, 1287–1292 (2012).

11.Marchler-Bauer, A. et al. CDD: NCBI's conserved domain database. *Nucleic Acids Res.* 43, D222–D226 (2014).

12. Röttig, M. et al. NRPSpredictor2—a web server for predicting NRPS adenylation domain specificity. *Nucleic Acids Res.* 39, W362–W367 (2011).

13. Vasil, Vimla, et al. Rapid production of transgenic wheat plants by direct bombardment of cultured immature embryos. *Nature Biotechnology* **11.12** 1553 (1993).

14. Russell, W. A. Registration of B70 and B73 Parental Lines of Maize1 (Reg. Nos. PL16 and PL17). *Crop Science* **12.5**, 721-721 (1972).

15. Schnable, P. S. et al. The B73 Maize Genome: Complexity, Diversity, and Dynamics. *Science* **326**, 1112–1115 (2009).

16. Zhang, L. et al. Identification and evaluation of resistance to Fusarium oxysporum f. sp. cubense tropical race 4 in Musa acuminata Pahang. *Euphytica* **214**, (2018).

17. Jin, Y. et al. Genome-wide profiling of sRNAs in the *Verticillium dahliae* -infected *Arabidopsis* roots. *Mycology* **9**, 155–165 (2018).

18. Yuan, T. L. et al. Optimization of transformation system of Fusarium graminearum. *Plant Physiol. Commun* **44**, 251–256 (2008).

19. Zhang, D. et al. The pollen receptor kinase LePRK2 mediates growth-promoting signals and positively regulates pollen germination and tube growth. *Plant Physiol.* **148**, 1368–1379 (2008).
