# Supplementary material for: A linear nonribosomal octapeptide from Fusarium graminearum facilitates cell-to-cell invasion of wheat
Source: Nat Commun. 2019 Feb 25;10:922. doi: 10.1038/s41467-019-08726-9 (PMC6389888; doi:10.1038/s41467-019-08726-9)
Supplement: Supplementary file 3 — Description of Additional Supplementary Files [file 41467_2019_8726_MOESM3_ESM.pdf]

Description of Additional Supplementary Files

File Name: Supplementary Data 1
Description: Fusaoctaxin A characterization data

File Name: Supplementary Data 2
Description: Expression data of wheat genes suppressed or induced by fusaoctaxin A

File Name: Supplementary Data 3
Description: Source data containing the raw data underlying all reported averages in graphs and charts and uncropped versions of gels and blots presented in this report.

File Name: Supplementary Data 4
Description: Chemical Structure nature template.cdx
