# Supplementary material for: A linear nonribosomal octapeptide from Fusarium graminearum facilitates cell-to-cell invasion of wheat
Source: Nat Commun. 2019 Feb 25;10:922. doi: 10.1038/s41467-019-08726-9 (PMC6389888; doi:10.1038/s41467-019-08726-9)
Supplement: Supplementary file 4 — Supplementary Data 1 [file 41467_2019_8726_MOESM4_ESM.doc]

**Supplementary Data 1. Fusaoctaxin A characterization data**

**A. Structure elucidation and stereochemistry determination**

Fusaoctaxin A was isolated as amorphous muds with the molecular formula C_36_H_68_N_8_O_10_ which deduced by the HR-ESI-MS pseudo-molecular ion peak of *m/z* 773.5135 [M+H]^+^ (cal. *m/z* 773.5131), corresponding to seven degree of unsaturation. The ^13^C NMR and HSQC spectra showed 36 carbon resonances, ascribed to eight carbonyl carbons, eleven methines (including seven connected with nitrogens), nine methylenes (three hydroxymethyls, one adjacent to an amino) and nine methyls. The ^1^H NMR spectra displayed seven amide NH proton doublet resonances (*δ*_H_ 8.11, 7.87, 8.07, 7.66, 7.94, 7.87, 7.43) in DMSO-*d*_6_ and seven typical *α* proton resonances (*δ*_H_ 4.41, 4.42, 4.36, 4.23, 4.30, 4.23, 3.74) of amino acid. The above data indicated that fusaoctaxin A should be an oligopeptide. The correlation of H-2/H-3/H-4 in ^1^H-^1^H COSY spectra, along with the HMBC correlation from H-2 to C-1 established a *γ*-amino butyl acid (GABA) moiety. The defielding effect of C-4 (*δ*_C_ 47.6) suggested a presence of a protonated amino adjacent to C-4. Further extensive analysis of 2D NMR (HSQC, ^1^H-^1^H COSY, HMBC) assigned another seven amino acids and the sequence (GABA_1_-Ala_2_-Ile_3_-Ser_4_-Val_5_-Ser_6_-Leu_7_-Leuol_8_) unambiguously (Supplementary Data 1C). The planar structure of the linear octapeptide was readily confirmed by the fragmentations of tandem MS (Supplementary Data 1L).

A combination analysis of modified Marfey’s method, NMR study and synthetic peptide comparison determined the stereochemistry of fusaoctaxin A. The compound was hydrolyzed by 6N HCl and then derivatized with Nα-(2,4-dinitro-5-fluorophenyl)-l-alaninamide (l-FDAA). The LCMS analysis of the reaction mixture with the authentic standards revealed Ser_4_, Val_5_, Ser_6_, Leu_7_ and Ile attributed to d configurations and Ala, Leuol attributed to l configurations. The further determination of whether *allo* forms of Ile or not was based on the ^1^H NMR coupling constant analysis and ROESY study. A large coupling constant of ^3^*J*_H-H_ = 5.3 Hz between H-9_Ile_ and H-10_Ile_ and the key REOSY correlations of H-10/15-NH, H-12/9-NH and H-9/H-13 indicated an *erythro* configuration for C-9 and C-10 (Supplementary Data 1C). Taken together, a d-*allo* configuration of Ile was hence established unambiguously. A detailed comparison (Supplementary Data 1B) of natural fusaoctaxin A NMR chemical shift (*δ* in ppm) with synthetic octapepetide with non-protonated confirmed the stereochemical structure as shown above.

B. ^1^H and ^13^C NMR Data of fusaoctaxin A and synthetic octapeptide

| Amino acid | Position | fusaoctaxin A^b^ | | Synthetic octapeptide^b^ | |
| --- | --- | --- | --- | --- | --- |
|  |  | *δ*_C_, type | *δ*_H_, mult. (*J* in Hz) | *δ*_C_, type | *δ*_H_, mult. (*J* in Hz) |
| GABA_1_ | 1 | 171.1, C |  | 171.2, C |  |
|  | 2 | 32.0, CH_2_ | 2.21, m | 31.9, CH_2_ | 2.21, t (7.3) |
|  | 3 | 21.0, CH_2_ | 1.80, m^a^ | 23.6, CH_2_ | 1.74, m |
|  | 4 | 47.6, CH_2_ | 2.93, m | 38.7, CH_2_ | 2.76, t (7.6) |
| l-Ala_2_ | 5 | 172.4, C |  | 172.6, C |  |
|  | 6 | 48.2, CH | 4.41, m^a^ | 48.3, CH | 4.40, m^a^ |
|  | 7 | 18.8, CH_3_ | 1.20, d (7.3) | 18.5, CH_3_ | 1.20, d (7.3) |
|  | 6-NH |  | 8.11, d (7.4) |  | 8.13, d (7.3) |
| d-allo-Ile_3_ | 8 | 171.2, C |  | 171.3, C |  |
|  | 9 | 55.1, CH | 4.42, dd (8.9, 5.3) | 55.1, CH | 4.42, dd (8.7, 5.5) |
|  | 10 | 37.3, CH | 1.83, m^a^ | 37.1, CH | 1.84, m |
|  | 11 | 25.8, CH_2_ | 1.31, m^a^  1.07, m | 25.8, CH_2_ | 1.30, m^a^  1.08, m |
|  | 12 | 14.3, CH_3_ | 0.76, d (6.9) | 14.3, CH_3_ | 0.77, d (6.9) |
|  | 13 | 11.6, CH_3_ | 0.82, m^a^ | 11.6, CH_3_ | 0.83, m^a^ |
|  | 9-NH |  | 7.87, d (8.9) |  | 7.85, d (8.7) |
| d-Ser_4_ | 14 | 170.0, C |  | 170.1, C |  |
|  | 15 | 55.1, CH | 4.36, dt (7.8, 6.4) | 55.2, CH | 4.37, dt (7.5, 6.0) |
|  | 16 | 61.4, CH_2_ | 3.59, d (6.4) | 61.4, CH_2_ | 3.60, d (6.0) |
|  | 15-NH |  | 8.07, d (7.8) |  | 8.06, d (7.8) |
| d-Val_5_ | 17 | 170.7, C |  | 170.7, C |  |
|  | 18 | 57.4, CH | 4.23, m^a^ | 57.5, CH | 4.20, m^a^ |
|  | 19 | 30.6, CH | 1.98, m | 30.5, CH | 1.99, m |
|  | 20 | 19.0, CH_3_ | 0.83, m^a^ | 19.0, CH_3_ | 0.84, m^a^ |
|  | 21 | 17.6, CH_3_ | 0.79, m^a^ | 17.7, CH_3_ | 0.80, m^a^ |
|  | 18-NH |  | 7.66, d (7.8) |  | 7.69, d (8.2) |
| d-Ser_6_ | 22 | 169.8, C |  | 169.8, C |  |
|  | 23 | 54.7, CH | 4.30, dt (7.8, 6.4) | 54.8, CH | 4.31, dt (7.8, 6.4) |
|  | 24 | 61.6, CH_2_ | 3.55, m^a^  3.50, m | 61.6, CH_2_ | 3.57, m^a^  3.49, dd (10.5, 6.9) |
|  | 23-NH |  | 7.94, d (7.8) |  | 7.96, d (7.8) |
| d-Leu_7_ | 25 | 171.3, C |  | 171.3, C |  |
|  | 26 | 51.3, CH | 4.23, m^a^ | 51.4, CH | 4.23, m^a^ |
|  | 27 | 40.6, CH_2_ | 1.45, m | 40.6, CH_2_ | 1.45, m |
|  | 28 | 24.2, CH | 1.60, m | 24.2, CH | 1.60, m |
|  | 29 | 23.1, CH_3_ | 0.85, m^a^ | 23.1, CH_3_ | 0.85, m^a^ |
|  | 30 | 21.4, CH_3_ | 0.80, m^a^ | 21.4, CH_3_ | 0.80, m^a^ |
|  | 26-NH |  | 7.87, d (8.2) |  | 7.88, d (8.2) |
| l-Leuol_8_ | 31 | 63.9, CH_2_ | 3.28, dd (10.5, 5.0)  3.17, dd (10.5, 6.9) | 63.9, CH_2_ | 3.28, dd (10.5, 5.0)  3.17, dd (10.5, 6.4) |
|  | 32 | 48.7, CH | 3.74, m | 48.7, CH | 3.75, m |
|  | 33 | 39.9, CH_2_ | 1.26, m | 39.9, CH_2_ | 1.26, m |
|  | 34 | 24.1, CH | 1.52, m | 24.1, CH | 1.52, m |
|  | 35 | 23.4, CH_3_ | 0.83, m^a^ | 23.4, CH_3_ | 0.83, m^a^ |
|  | 36 | 21.7, CH_3_ | 0.78, m^a^ | 21.7, CH_3_ | 0.78, m^a^ |
|  | 32-NH |  | 7.43, d (8.7) |  | 7.44, d (8.7) |
| ^a^ overlap; ^b^ recorded at 500 MHz of ^1^H NMR and 125 MHz of ^13^C NMR in DMSO-d6 | | | | | |

C. Key correlations of fusaoctaxin A: a, correlations of ^1^H-^1^H COSY and HMBC; b, Newman projection and ROESY correlations of d-*allo*-Ile

D. Extracted Ion Chromatogram (EIC, m/z 342, 358, 370,384) of l-FDAA derivatives of fusaoctaxin A hydrolysate and those of d- or l- amino acid authentic standards

**E. ^13^C NMR of fusaoctaxin A in DMSO-*d*_6_ (125 MHz)**

**F. ^1^H NMR spectrum of fusaoctaxin A in DMSO-*d*_6_ (500 MHz)**

**G. HSQC spectrum of fusaoctaxin A**

**H. ^1^H-^1^H COSY spectrum of fusaoctaxin A**

**I. HMBC spectrum of fusaoctaxin A**

**J. ROESY spectrum of fusaoctaxin A**

**K. HR-ESI-MS spectrum of fusaoctaxin A**


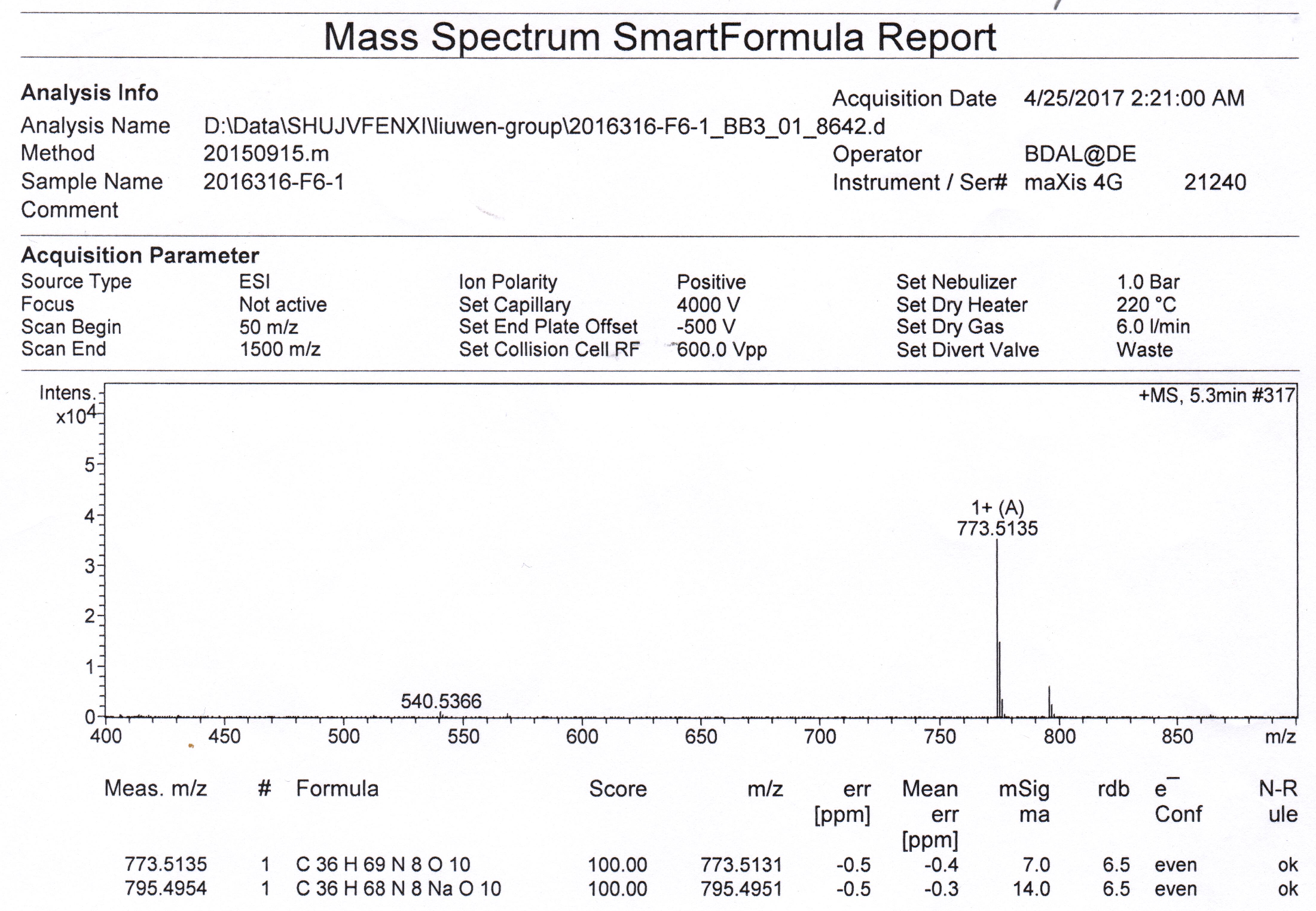


**L. Tandem MS fragmentation of fusaoctaxin A**

**M. ^13^C NMR of synthetic octapeptide in DMSO-*d*_6_ (125 MHz)**

**N. ^1^H NMR spectrum of synthetic octapeptide in DMSO-*d*_6_ (500 MHz)**
