# Supplementary material for: A linear nonribosomal octapeptide from Fusarium graminearum facilitates cell-to-cell invasion of wheat
Source: Nat Commun. 2019 Feb 25;10:922. doi: 10.1038/s41467-019-08726-9 (PMC6389888; doi:10.1038/s41467-019-08726-9)
Supplement: Supplementary file 8 — Reporting Summary [file 41467_2019_8726_MOESM8_ESM.pdf]

nature research

Corresponding author(s): Weihua Tang, Wen Liu

Last updated by author(s): Jan 11, 2019

Reporting Summary

Nature Research wishes to improve the reproducibility of the work that we publish. This form provides structure for consistency and transparency in reporting. For further information on Nature Research policies, see Authors & Referees and the Editorial Policy Checklist.

Statistics

For all statistical analyses, confirm that the following items are present in the figure legend, table legend, main text, or Methods section.

n/a	Confirmed	
☐	☒	The exact sample size (*n*) for each experimental group/condition, given as a discrete number and unit of measurement
☐	☒	A statement on whether measurements were taken from distinct samples or whether the same sample was measured repeatedly
☐	☒	The statistical test(s) used AND whether they are one- or two-sided<br>*Only common tests should be described solely by name; describe more complex techniques in the Methods section.*
☒	☐	A description of all covariates tested
☒	☐	A description of any assumptions or corrections, such as tests of normality and adjustment for multiple comparisons
☐	☒	A full description of the statistical parameters including central tendency (e.g. means) or other basic estimates (e.g. regression coefficient) AND variation (e.g. standard deviation) or associated estimates of uncertainty (e.g. confidence intervals)
☐	☒	For null hypothesis testing, the test statistic (e.g. *F*, *t*, *r*) with confidence intervals, effect sizes, degrees of freedom and *P* value noted<br>*Give P values as exact values whenever suitable.*
☒	☐	For Bayesian analysis, information on the choice of priors and Markov chain Monte Carlo settings
☒	☐	For hierarchical and complex designs, identification of the appropriate level for tests and full reporting of outcomes
☒	☐	Estimates of effect sizes (e.g. Cohen's *d*, Pearson's *r*), indicating how they were calculated

Our web collection on statistics for biologists contains articles on many of the points above.

Software and code

Policy information about availability of computer code

Data collection

Provide a description of all commercial, open source and custom code used to collect the data in this study, specifying the version used OR state that no software was used.

Data analysis

Provide a description of all commercial, open source and custom code used to analyse the data in this study, specifying the version used OR state that no software was used.

For manuscripts utilizing custom algorithms or software that are central to the research but not yet described in published literature, software must be made available to editors/reviewers. We strongly encourage code deposition in a community repository (e.g. GitHub). See the Nature Research guidelines for submitting code & software for further information.

Data

Policy information about availability of data

All manuscripts must include a data availability statement. This statement should provide the following information, where applicable:

- Accession codes, unique identifiers, or web links for publicly available datasets
- A list of figures that have associated raw data
- A description of any restrictions on data availability

All data and genetic materials used for this paper are available from the authors upon reasonable request.

Field-specific reporting

Please select the one below that is the best fit for your research. If you are not sure, read the appropriate sections before making your selection.

☒ Life sciences ☐ Behavioural & social sciences ☐ Ecological, evolutionary & environmental sciences

Life sciences study design

All studies must disclose on these points even when the disclosure is negative.

Sample size	We repeated three or more independent experiments.<br>The previous research and detailed protocol publication below showed low variation under controled conditions:<br>Jia, L.-J., Wang, W.-Q. & Tang, W.-H. Wheat Coleoptile Inoculation by Fusarium graminearum for Large-scale Phenotypic Analysis. Bio-Protocol 7 (2017)
Data exclusions	No data were excluded.
Replication	At least three replicates were repeated and all the three results were reproducible to support a conclusion.
Randomization	Not randomized. But repeated on different days.
Blinding	For assaying activity of purified octapeptide, double blinded experiments were performed. One author H.T. labeled the fractions with and with out the purified octapeptide with numbers, another author Q. Wang performed infection assays with addition of this fractions, and measured lesion sizes before knowing the meanings of numbers. Other experiments were not blinded.

Reporting for specific materials, systems and methods

We require information from authors about some types of materials, experimental systems and methods used in many studies. Here, indicate whether each material, system or method listed is relevant to your study. If you are not sure if a list item applies to your research, read the appropriate section before selecting a response.

Materials & experimental systems

n/a	Involved in the study	
☒	☐	Antibodies
☐	☒	Eukaryotic cell lines
☒	☐	Palaeontology
☒	☐	Animals and other organisms
☒	☐	Human research participants
☒	☐	Clinical data

Methods

n/a	Involved in the study	
☒	☐	ChIP-seq
☒	☐	Flow cytometry
☒	☐	MRI-based neuroimaging

Eukaryotic cell lines

Policy information about cell lines

Cell line source(s)	The GeneScript Co. (Nanjing, China) performed the experiments and the cell lines were bought from ATCC (American Type Culture Collection).
Authentication	Yes, the cell lines were authenticated following the standard for biological reagent and assay quality set by ATCC.
Mycoplasma contamination	We have examined. None of cell lines used in this paper were contaminated with mycoplasma.
Commonly misidentified lines<br>(See ICLAC register)	None of cell lines used in this paper listed in the database of commonly misidentified cell lines maintained by ICLAC.
